# An Experimental and Computational Evolution-Based Method to Study a Mode of Co-evolution of Overlapping Open Reading Frames in the AAV2 Viral Genome

**DOI:** 10.1371/journal.pone.0066211

**Published:** 2013-06-24

**Authors:** Yasuhiro Kawano, Shane Neeley, Kei Adachi, Hiroyuki Nakai

**Affiliations:** 1 Department of Molecular and Medical Genetics, Oregon Health and Science University School of Medicine, Portland, Oregon, United States of America; 2 Takara Bio Inc., Otsu Shiga, Japan; Mayo Clinic, United States of America

## Abstract

Overlapping open reading frames (ORFs) in viral genomes undergo co-evolution; however, how individual amino acids coded by overlapping ORFs are structurally, functionally, and co-evolutionarily constrained remains difficult to address by conventional homologous sequence alignment approaches. We report here a new experimental and computational evolution-based methodology to address this question and report its preliminary application to elucidating a mode of co-evolution of the frame-shifted overlapping ORFs in the adeno-associated virus (AAV) serotype 2 viral genome. These ORFs encode both capsid VP protein and non-structural assembly-activating protein (AAP). To show proof of principle of the new method, we focused on the evolutionarily conserved QVKEVTQ and KSKRSRR motifs, a pair of overlapping heptapeptides in VP and AAP, respectively. In the new method, we first identified a large number of capsid-forming VP3 mutants and functionally competent AAP mutants of these motifs from mutant libraries by experimental directed evolution under no co-evolutionary constraints. We used Illumina sequencing to obtain a large dataset and then statistically assessed the viability of VP and AAP heptapeptide mutants. The obtained heptapeptide information was then integrated into an evolutionary algorithm, with which VP and AAP were co-evolved from random or native nucleotide sequences *in silico*. As a result, we demonstrate that these two heptapeptide motifs could exhibit high degeneracy if coded by separate nucleotide sequences, and elucidate how overlap-evoked co-evolutionary constraints play a role in making the VP and AAP heptapeptide sequences into the present shape. Specifically, we demonstrate that two valine (V) residues and β-strand propensity in QVKEVTQ are structurally important, the strongly negative and hydrophilic nature of KSKRSRR is functionally important, and overlap-evoked co-evolution imposes strong constraints on serine (S) residues in KSKRSRR, despite high degeneracy of the motifs in the absence of co-evolutionary constraints.

## Introduction

Viral genes often share a potion of their nucleotide sequence to make overlapping open reading frames (ORFs). Having an alternative ORF in one gene is a tactic for viruses to accommodate a necessary quantity of genetic information within a limited capacity of their genomes [1]. In such genomic organization, a synonymous mutation or a beneficial non-synonymous mutation in one ORF may result in a deleterious amino acid change in the overlapping ORF. Therefore, overlapping ORFs must undergo co-evolution in a sophisticated manner. How viral overlapping ORFs emerged, evolved, and became fixed under strong overlap-evoked co-evolutionary constraints has been a compelling question. To date, this question has been addressed primarily by homologous sequence alignment studies. Keese and Gibbs have proposed a mechanism called overprinting, by which new mutations are introduced in a pre-existing ORF, giving birth to a second ORF therein [2]. Subsequent studies have also lent strong support to the idea that overprinting is a mechanism for the origination of many viral overlapping genes [3], [4], [5], [6]. This evolutionary process has been shown to result in codon usage bias, reduced synonymous amino acid substitutions, higher contents of six-fold degenerate amino acid residues, and higher degrees of conservation associated with slower evolution [7], [8], [9]. The identification of such molecular signatures has led to discovery of new overlapping ORFs in viral genomes [8], [10], [11]. However, the fundamental question still remains as to how co-evolutionary constraints play roles at individual amino acid levels in making newly emerging overlapping ORFs into their present shapes. Despite the prevalence of overlapping ORFs in viral genomes, such a question is difficult to address by conventional sequence alignment approaches, particularly when no evolutionary diversity of amino acids coded by shared nucleotides is found in either of the overlapping ORFs.

Adeno-associated virus (AAV) is a promising gene delivery vehicle for human gene therapy [12], [13], [14], [15]. It was recently discovered that the viral genomes of various AAV serotypes contain a frame-shifted alternative ORF of approximately 0.6 kb in length completely embedded within the 2.2-kb ORF coding the capsid VP protein [16], [17]. This second ORF encodes the non-structural assembly-activating protein (AAP), a nucleolar protein indispensable for virion assembly [16], [17]. AAP transports VP to the nucleoli where the VP proteins assemble into capsids. This transport is mediated by the VP-AAP interaction through a hydrophobic amino acid-rich domain in the N-terminus of AAP and the 2-fold symmetry axis region in the capsid composed of amino acids near the C-terminus of VP [18]. In addition, AAP induces conformational changes of VP monomers and oligomers to promote capsid assembly [18]. Such new knowledge about AAP has furthered our understanding of the mechanism involved in AAV capsid assembly. In the meantime, the discovery of the VP/AAP-overlapping ORFs has raised a possibility that some or many of the evolutionarily highly conserved amino acids in VP and AAP within the overlapping regions are conserved not due to structural and/or functional significance of these amino acids themselves, but due to co-evolutionary constraints imposed by structural and/or functional constraints of the superimposing amino acids in the overlapping ORF protein product. This VP/AAP overlap confounds the interpretation of evolutionary conservation of each amino acid in the VP and AAP proteins.

Here we report a new methodology that overcomes the above-mentioned limitation of the conventional homologous sequence alignment approaches in interpreting the nature of amino acid conservation in proteins coded by viral overlapping ORFs. The new method exploits experimental directed evolution [19], [20], [21], [22], Illumina sequencing [23] and evolutionary computing [24]. To show proof-of-principle of this method, we focused on a particular set of evolutionarily conserved overlapping VP and AAP heptapeptide motifs, QVKEVTQ and KSKRSRR, coded by the AAV serotype 2 (AAV2) VP and AAP ORFs, respectively. AAV2 is the prototype AAV that was first isolated and has been most extensively studied for viral structure, function and life cycle; therefore, AAV2 was used for this study. As a result, we could successfully elucidate the significance of conservation of individual amino acid residues in the evolutionarily conserved heptapeptide motifs in VP and AAP, and gain insights into how overlapping VP and AAP ORFs in the AAV2 genome emerged and have co-evolved at individual amino acid levels.

## Results

### The N-terminal Two-fifths of the VP3 Protein is Highly Conserved: what has Imposed Evolutionary Constraints?

**Figure 1:**
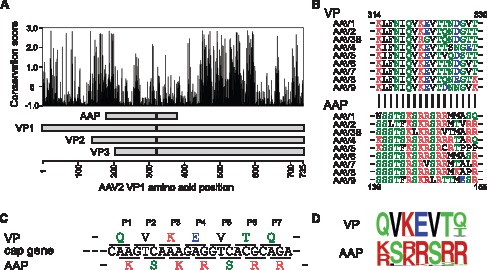
The VP/AAP overlapping ORFs in the AAV2*cap* gene. (**A**) Organization of the *cap* gene and evolutionary conservation of the VP proteins. Evolutionary conservation scores were calculated by a ConSurf analysis [39] of 128 AAV species. (**B**) Sequence alignment of amino acids around the VP and AAP overlapping regions indicated with black lines in Panel A. The QVKEVTQ/KSKRSRR motifs are indicated with seven thick vertical lines. The numbers indicate amino acid positions. (**C**) VP/AAP-overlapping ORFs encoding the QVKEVTQ/KSKRSRR motifs. (**D**) Amino acid sequence logos (frequency plots) representing the VPs and AAPs derived from 128 AAV species.

A sequence alignment analysis of VP proteins of 128 AAVs isolated from various animal species reveals that many amino acids in the VP protein within the amino (N)-terminal two-fifths of the VP3 protein are evolutionarily highly conserved (**Figure 1A**). Until the discovery of AAP, the strong evolutionary conservation in this region was deemed to be attributed primarily to the structural constraints imposed by the core structure of the VP protein including the β-barrel [25]. In this respect, the discovery of AAP has challenged the traditional view of the mechanism of the amino acid conservation in this region. When co-evolution of VP and AAP is taken into account, a question arises as to which of structural, functional and overlap-evoked co-evolutionary constraints restrict the diversity of amino acids at each position within this overlapping region of the VP protein. This question is difficult to address by conventional approaches, but could be answered by the new method reported here.

### The QVKEVTQ (in VP)/KSKRSRR (in AAP) Motifs: the Highly Conserved Overlapping Heptapeptides Focused on in the Study

To show proof of principle of the new method, we chose a pair of overlapping VP/AAP motifs of 7 amino acids that are both evolutionarily highly conserved. By aligning 128 known and putative AAP proteins, we identified a well conserved basic amino acid-rich motif, (K/R)S(K/R)RSRR (note: KSKRSRR in the case of AAV2 AAP) (**Figure 1B** and **D**). QVKEVTQ, the AAV2 VP heptapeptide translated from the nucleotides coding KSKRSRR, also exhibits a high degree of conservation except for the 7th amino acid position (**Figure 1B** and **D**). Thus we selected the QVKEVTQ/KSKRSRR motifs (**Figure 1C**) in the subsequent proof of principle experiments.

### Experimental Evolution of the Overlapping VP and AAP Proteins without Co-evolutionary Constraints

**Figure 2:**
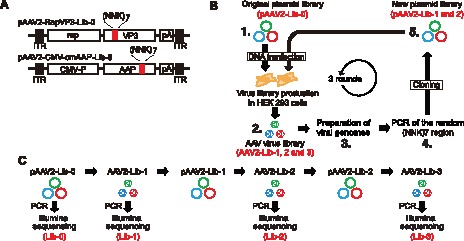
The approach used for the experimental directed evolution of the VP/AAP-overlapping ORFs. (**A**) Map of VP3 and AAP mutant plasmid libraries. Red bars indicate the random heptapeptide region corresponding to the QVKEVTQ/KSKRSRR motifs. (**B**) Schematic representation of the AAV directed evolution procedure. The numbers 1 through 5 in the figure indicate steps in the procedure. Step 1, Construction of the original plasmid DNA library (pAAV2-Lib-0); Step 2, Production of AAV virus library (AAV2-Lib-1); Step 3, Recovery of viral genomic DNA; Step 4, PCR amplification of the heptapeptide-coding region in the viral genome; Step 5, Construction of the next round plasmid DNA library using the PCR amplicons from Step 4 (pAAV2-Lib-1). Then Step 2 follows for the next round positive selection. This procedure was repeated three times to obtain AAV2-Lib-2 and AAV2-Lib-3. (**C**) The timeline of Illumina sequencing of PCR amplicons of the heptapeptide region. Illumina sequencing was used for extensively characterize the heptapeptides evolved by the experimental procedure.

What amino acid sequences would the overlapping VP and AAP regions take if the two regions were coded by separate nucleotide sequences? We first addressed this question. To this end, we took an experimental directed evolution approach in the absence of overlap-evoked co-evolutionary constraints, and identified many viable mutants of each of the QVKEVTQ and KSKRSRR motifs in the VP/AAP-overlapping ORFs by Illumina sequencing. Such overlap-evoked co-evolutionary constraint-free evolution of VP and AAP was possible by taking a transcomplementation approach [16]. We constructed two AAV plasmid libraries, pAAV2-RepVP3-Lib-0 and pAAV2-CMV-cmAAP-Lib-0 with the diversity of 2×10^6^ (**Figure 2A** and Step 1 in **Figure 2B**). The numbers at the end of the plasmid names indicate the number of rounds of selection. In these libraries, the 21-nucleotide-long sequence in the AAV2 viral genome corresponding to QVKEVTQ in VP3 ORF or that corresponding to KSKRSRR in AAP ORF was replaced with random 21-mer nucleotides. The AAV2-RepVP3 genome expresses all the AAV Rep proteins and VP3 protein, but does not express VP1, VP2 or AAP protein. To make AAV2-RepVP3 viral particles, wild type codon-modified (cm) AAP (cmAAP) was provided in trans (*i.e.*, transcomplementation). The AAV2-CMV-cmAAP genome expresses only cmAAP. In the directed evolution procedure for VP3, we transfected HEK293 cells with pAAV2-RepVP3-Lib-0 together with helper plasmids to produce AAV2-RepVP3-Lib-1 (Step 2 in **Figure 2B**). We then recovered viral genomes from nuclease-resistant particles (Step 3 in **Figure 2B**), PCR-amplified the random 21-mer nucleotide regions in VP (Step 4 in **Figure 2B**), and cloned the PCR amplicons into the AAV plasmid library backbone, pAAV2-RepVP3-Lib-BB, to create the next round plasmid library, pAAV2-RepVP3-Lib-1 (Step 5 in **Figure 2B**). We repeated this procedure three times until we obtained AAV2-RepVP3-Lib-3 viral particles. PCR amplicons of the random 21-mer nucleotide regions in each cycle were sequenced on the Illumina platform to obtain a large set of information about viable mutants (**Figure 2C**). The directed evolution of AAP was performed in the same manner until we obtained AAV2-CMV-cmAAP-Lib-3 viral particles. The selective pressure given in the procedure was limited to the ability to form nuclease resistant VP3-only intact particles with viral genomes packaged.

### Identification of viable QVKEVTQ VP3 Mutants by Experimental Evolution

**Figure 3:**
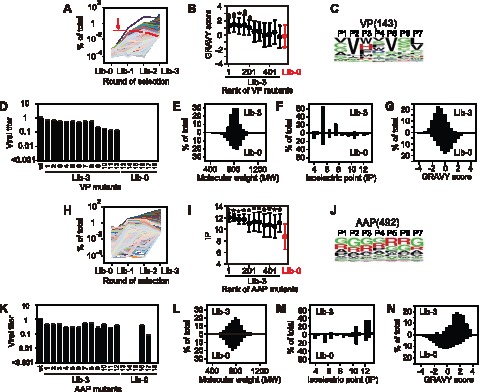
The results of the experimental evolution of VP and AAP heptapeptides and their biochemical characterization. (**A** and **H**) Evolutionary history of the viable VP (Panel A) and AAP (Panel H) heptapeptide mutants. Percentage of sequence reads of each mutant among all the sequence reads is plotted as a function of the number of selection cycles. The red line with an arrow in Panel A represents GGGGGGG. (**B** and **I**) Chemical properties of top-ranked VP (Panel B) and AAP (Panel I) heptapeptides with no stops identified in Lib-3. Top-ranked 550 heptapeptides are divided into 11 bins (50 peptides per bin) according to their rank. Red lines indicate chemical properties representing all the 930372 VP heptapeptides and 554743 AAP heptapeptides in Lib-0. Asterisks indicate statistical significance with *P*<0.05 and power >0.8 (Mann Whitney U-test) when compared to the values of Lib-0. The power analysis was performed by comparing 50 heptapeptides in each bin with 50 randomly selected heptapeptides from Lib-0 100 times. Vertical bars represent standard deviations. (**C** and **J**) Amino acid frequency plots of the 143 viable VP heptapeptide mutants (Panel C) and the 492 viable AAP heptapeptide mutants (Panel J) identified by the experimental evolution of random nucleotide sequences. (**D** and **K**) Experimental validation of the capsid-forming ability of the viable VP (Panel D) and AAP (Panel K) heptapeptides identified by the experimental directed evolution procedure. In Panel D, relative titers of wild type and mutant AAV2 VP3 only viral particles recovered from one 6 cm dish are shown. The VP heptapeptide sequences selected from the viable 143 VP heptapeptide mutants found in Lib-3 (*i.e.*, 1 through 12) and those selected from the random mutants found in Lib-0 (*i.e.*, 13 through 18) are as follows: 1, SAYWVTQ; 2, TVWASSV; 3, CLHDVMS; 4, SVHDACV; 5, GVFWVGV; 6, VVHDVSD; 7, CVRDVTL; 8, CVWSVGL; 9, 10, QVWDERY; 11, AVMTVCS; 12, CVFFSSF; 13, PHNFVAL; 14, LDDFLEF; 15, EGPCGGL; 16, RGAEWNK; 17, GGRWGRG; 18, GVAWGVG. In Panel K, relative titers of wild type AAV2 VP3 only viral particles produced with wild type or each mutant AAP in one 6 cm dish are shown. The AAP heptapeptide sequences in the AAP mutants selected from Lib-3 (*i.e.*, 1 through 12) and Lib-0 (*i.e.*, 13 through 18) are as follows: 1, GGGRRRR; 2, RGRRRRW; 3, VRRRRGG; 4, WRRPRRV; 5, PRLSRRR; 6, APGRGAR; 7, RGGRRRA; 8, GRVGPRG; 9, RRVGRLG; 10, PGRGRRG; 11, VGGGGRR; 12, GERKGRG; 13, RSSPALR; 14, PGGGSIS; 15, GAQVGVV; 16, GGARRGG; 17, RGHDGAS; 18, ACWRLF_ (a stop codon follows after F). All the experiments were performed in triplicate. (**E, F, G, L, M,** and **N**) Histograms showing chemical properties (MW, IP and GRAVY score) of the viable VP (Panels E, F and G) and AAP mutants (Panels L, M and N). In each graph, upper bars represent histograms of the viable 143 VP or 492 AAP heptapeptide mutants found in Lib-3 and lower bars represent histograms of 930372 VP and 554743 AAP heptapeptides found in Lib-0.

Three rounds of selection of the AAV2-RepVP3-Lib for viable AAV particle formation identified many potentially viable VP3 mutants. When we ranked the identified heptapeptide mutant clones according to their sequence read numbers obtained by Illumina sequencing, the highest rank of the clones with one or more stop codons within the heptapeptide regions in the library Lib-3 was 145 and all the top-ranked 144 clones had no stops. To investigate whether or not a majority of the 144 clones, if not all, represent viable mutant clones positively selected by the directed evolution procedure, we took 5 independent approaches as follows. First, we tracked evolutional history of the positively selected clones. We could track evolutional history in 98 of the 144 clones and found that the GGGGGGG clone ranked 144 in the library Lib-3 showed steady extinction (**Figure 3A**), which was then excluded. Second, the null hypothesis that the top-ranked 143 clones with no stops represent a pool of clones that were randomly selected from the library, was rejected by taking into account the frequency of clones with a stop(s) in the library Lib-0, which was found to be 15.4% (*P* = 3.7×10^−11^, binomial test). Third, as detailed in the following section, biochemical characterization of the heptapeptides revealed that the clones ranked 250 or higher, including the top-ranked 143 clones, were significantly more hydrophobic than the clones in the library Lib-0 (*P* = 4.9×10^−12^ to 6.1×10^−6^, Mann-Whitney U-test) (**Figure 3B**). Fourth, the amino acid composition of the top-ranked 250 clones, including the 143 clones, was significantly different from that in the library Lib-0 (*P = *0.000011, Mann-Whitney U-test, Tables S1, S2 and S3). The amino acid compositions of the top-ranked 143 mutants are shown as a frequency plot in **Figure 3C**. Finally, we experimentally verified the viability of 12 of the 143 clones by quantifying virus production yield in HEK293 cells. All the 12 mutants were viable while none of the 6 clones from the library Lib-0 produced viral particles at a detectable level (**Figure 3D**). Thus, this experiment not only provided amino acid sequence information of capsid-forming viable heptapeptide VP mutants but also demonstrated that our approach using experimental evolution, Illumina sequencing and biostatistics successfully identifies many viable mutants without experimental validation.

### High Degeneracy Albeit Conservedβ-strand-forming Propensity of the QVKEVTQ Motif under no Co-evolutionary Constraints

**Figure 4:**
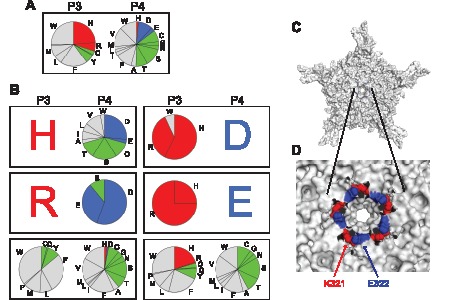
Peculiar positive-negative and neutral-neutral amino acid combinations at P3 and P4 in the 143 viable VP heptapeptides. (**A**) Amino acid compositions of all the 143 viable VP heptapeptide mutants. (**B**) Various amino acid combinations at P3 and P4 positions in the viable VP heptapeptide mutants. Left panels show P3/P4 combinations when histidine (H, upper left), arginine (R, middle left) or a non-charged amino acid (lower left) is at P3. Right panels show P3/P4 combinations when aspartic acid (D, upper right), glutamic acid (E, middle right) or a non-charged amino acid (lower right) is at P4. (**C**) Topological location of amino acid residues at P3 (K321, red) and P4 (E322, blue) in the wild type AAV2 capsid VP3 is shown on a VP3 pentamer viewed down an icosahedral five-fold symmetry axis at the center. E322 is partially exposed on the outer surface near the five-fold pore while K321 is barely seen from the outside of the capsid. (**D**) A close-up view of K321 and E322. Ten amino acids (from V323 to I332) forming the outer ridge around the five-fold pore are removed to make K321 and E322 visible. Five pairs of K321 and E322 form a ring in the five-fold channel wall. Panels C and D are created using PyMOL.

The top-ranked 143 peptides were all heptapeptides and were used to extract the information about the following biochemical properties; (1) amino acid compositions (**Table S1**); (2) molecular weights (**Figure 3E**); (3) isoelectric points (**Figure 3F**); and (4) hydropathicity deduced by the grand average of hydropathicity (GRAVY) scores [26] (**Figure 3G**). We found that hydrophobic valine (V) residues at P2 and P5 are well conserved in the viable VP heptapeptide mutants (**Figure 3C**). At P3, histidine (H), phenylalanine (F), tryptophan (W) and tyrosine (Y), which share a similar stereochemical property, account for 75% (**Figure 4A**). Lysine (K), the highly conserved amino acid at P3 in nature, was completely excluded from the position. Interestingly, amino acids at P3 and P4 exhibit a peculiar positive-negative or neutral-neutral combination (**Figure 4B**). This makes the net charge of these two consecutive amino acids exclusively neutral. In the wild type, residues at P3 and P4 are K321 and E322, respectively, and 5 pairs of K321 and E322 residues from 5 different VP subunits are juxtaposed with each other forming a ring along the wall inside the pore at the five-fold symmetry axis (**Figure 4C** and **D**). This indicates a structural role of the net neutral charge of this five-fold axis ring comprising 10 amino acid residues in maintaining the stability of the five-fold axis pores. Despite several conserved features described above, the QVKEVTQ motif exhibits high amino acid degeneracy except for P2 and P5 (**Figure 3C**).

**Figure 5:**
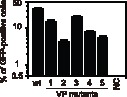
HEK293 cell transduction with wild type AAV2 and viable AAV2 VP heptapeptide mutants. HEK293 cells were infected with dsAAV2-CMV-GFP encapsidated with wild type or mutant capsid at MOI of 20,000. The mutant capsids carried the following VP heptapeptide mutants: 1, SAYWVTQ; 2, TVWASSV; 3, CLHDVMS; 4, SVHDACV; and 5, GVFWVGV. Forty-eight hours post-infection, percentage of GFP-positive cells was determined by flow cytometry. Non-transduced cells were used as a negative control (NC). Vertical bars represent standard deviations.

In addition to the biochemical properties of the primary structure of the viable VP heptapeptides, we also investigated their secondary structures using the Discrimination of Secondary Structure Class (DSC) method [27]. In the wild type QVKEVTQ heptapeptide sequence, the amino acid residues at P1, P2, P3 and P4 (*i.e.*, QVKE) form a β-strand while those at P5, P6 and P7 exhibit a coil that does not have a regular secondary structure [25]. The QVKE residues are the carboxy terminal four amino acids in the longest β-strand among the 8 β-strands composing the core β-barrel. In concordance with this, the DSC predicts that QVKEVT residues at P1 to P6 form a β-strand. This property of β-strand formation was not clear in 930372 random VP heptapeptides in the library Lib-0 (**Figure S1A**) or computer-generated 10000 random VP heptapeptides (**Figure S1I**) but was prominent in the 143 viable VP heptapeptides (**Figure S1B**). A simulation study revealed that having a valine (V) residue at either P2 or P5 is sufficient to form a β-strand in the milieu of heptapeptide motifs composed of random amino acid sequences (**Figure S1J, K** and **L**). This strong ability of the P2 residue to direct β-strand formation irrespective of the type of the other six amino acids was retained in other hydrophobic amino acids such as isoleucine (I) but was abolished in hydrophilic amino acids such as asparagine (N) (**Figure S1M** and **N**). Twenty-one out of 143 viable VP heptapeptides did not have valine (V) residues at either P2 or P5 but still exhibited strong propensity to form a β-strand (**Figure S1O**). These observations indicate that β-strand formation of the heptapeptide motif is the most important biochemical property for viral particle formation that should be conserved; however, diverse types of amino acid sequences can fulfill this requirement, resulting in high degeneracy of the QVKEVTQ motif under no co-evolutionary constraints. The high degree of degeneracy of the amino acid sequence did not abolish infectivity because 5 viable mutants exhibiting degenerate amino acid sequences all infected and transduced HEK293 cells (**Figure 5**).

### Identification of viable KSKRSRR AAP Mutants by Experimental Evolution

To identify viable mutants alternative to the KSKRSRR motif in AAP, we performed a reciprocal experiment using the AAV2-CMV-cmAAP-Lib library in the same manner as that for AAV2-RepVP3-Lib library (**Figure 2**). The highest rank of the clones with a stop(s) in the library Lib-3 was 498, and the frequency of clones with a stop(s) in the library Lib-0 was 7.1%, which makes it impossible to explain the exclusion of clones with a stop(s) from the pool of the top-ranked 497 clones by a random model (*P = *1.3×10^−16^, binomial test). Among the 497 mutants, 492 were heptapeptides. We could track evolutional history in 484 clones and found none of them showed steady extinction in the experimental evolution process (**Figure 3H**). In addition, the 492 heptapeptides represent a population comprising peptides of strongly basic nature compared to the heptapeptides in the library Lib-0 (*P*<0.05, power = 1.0, Mann-Whitney U-test) (**Figure 3I**) due primarily to the high frequency of arginine residues (**Figure 3J**). Moreover, the amino acid composition of the top-ranked 550 clones was significantly different from that in the library Lib-0 (*P* = 0.000011 to 0.049860, Mann-Whitney U-test, Table S3). Furthermore, 12 AAV2-CMV-cmAAP mutants selected from the top-ranked 492 clones were all viable and generated AAV particles efficiently, while only 2 of 5 mutants from the non-selected library Lib-0 excluding the one with a stop codon retained the AAP function (**Figure 3K**). These observations provide evidence that the top-ranked majority of the 492 mutant clones, if not all, are functionally competent and represent a pool of AAP mutants that have the ability to exert their capsid assembly function.

### Greater Degeneracy of the KSKRSRR Motif under no Co-evolutionary Constraints

The biochemical information of the heptapeptide region of the viable AAP mutants was collected from the top-ranked 492 peptides in the same way as that for the VP peptides. The top-ranked heptapeptides are strongly basic and exhibit hydrophilicity (**Figure 3L, M** and **N**). Unlike the QVKEVTQ region in VP, no remarkable position-dependent difference in the amino acid compositions was observed (**Figure 3J** and **Table S1**). At all seven positions, arginine (R) and glycine (G) were preferably found. On the other hand, hydrophobic isoleucine (I), leucine (L), methionine (M), phenylalanine (F), tryptophan (W) and valine (V) were negatively selected (**Tables S1** and **S2**). There was no difference in the secondary structure profiles between 554743 random AAP heptapeptides in the library Lib-0 (**Figure S1E**) and the 492 viable AAP heptapeptides (**Figure S1F**); and no regular secondary structure was prominent. Taken together, the data demonstrate that positive charge and hydrophilicity are the most important biochemical features and it is not important how each amino acid is arranged.

### Computational Co-evolution of VP/AAP

**Figure 6:**
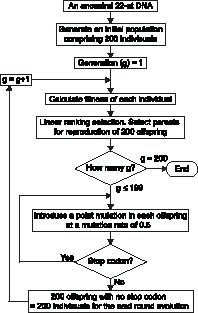
A flowchart of the evolutionary algorithm. This flowchart outlines the steps involved in the computational directed evolution of 22 nucleotide-long DNA coding the VP/AAV-overlapping heptapeptides.

**Figure 7:**
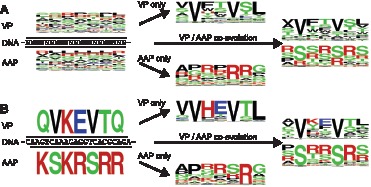
Amino acid frequency plots of computationally evolved VP and AAP heptapeptides. (**A**) Computational evolution of random nucleotide sequences. The *in silico* evolution was performed in three ways, VP only evolution without AAP-originating constraints, AAP only evolution without VP-originating constraints, and VP and AAP co-evolution. The amino acid logos to the left are a representative set of 200 ancestral sequences. The logos in the middle and to the right represent amino acid frequencies in all the individuals in the 200th generation. (**B**) The same analysis was done using the wild type AAV2 genome sequence.

To understand the co-evolution of VP and AAP, we developed an evolutionary algorithm (**Figure 6**) into which we integrated the experimentally elucidated constraints on each of the VP/AAP-overlapping ORFs as described in Materials and Methods and detailed in **Text S1**. We first evolved the overlapping heptapeptide region *in silico* from random 22-mer nucleotides under the constraints imposed by either VP or AAP only. The sequence frequency logos representing the amino acid compositions of the computationally evolved heptapeptide regions were found to be quite similar to those of the experimentally evolved top-ranked peptides (**Figures 3C** and **7A**). In addition, the unique pattern of secondary structure propensities observed in the viable heptapeptides could be reproduced in the *in silico*-evolved heptapeptides (**Figure S1C, D, G, H** and **P**). These observations validated our approach to the *in silico* evolution of the VP/AAP-overlapping ORFs.

Next, we co-evolved the overlapping heptapeptide region *in silico* from random 22-mer nucleotides. The most remarkable finding was the amino acid composition change in AAP. In all 7 amino acid positions in the co-evolved AAP ORFs, serine (S) and arginine (R) residues were the two most frequently found amino acids (**Figure 7A**). This results in the exhibition of the (R/S)_7_ pattern with amino acids at P2 and P5 being serine (S) residues in more than the half of the heptapeptides evolved *in silico*. This recapitulates well the sequence found in nature; *i.e.*, (K/R)S(K/R)RSRR. The VP heptapeptide sequence was also influenced, though to a lesser degree, resulting in some changes in the amino acid compositions. At P3, a basic amino acid histidine (H), which was found in 24% and 20% of experimentally and computationally evolved VP heptapeptides, respectively, was eliminated during the computational co-evolution. Leucine (L) was the major amino acid found at P7 in the VP heptapeptide under single evolution, but it became less favored under co-evolution. These observations provide insights into how overlap-evoked constraints play roles in the co-evolution of the VP/AAP-overlapping ORFs.

Finally, we investigated co-evolution of VP and AAP by computationally evolving the VP/AAP-overlapping heptapeptide ORFs found in nature, QVKEVTQ/KSKRSRR, independently or under co-evolutionary constraints. In single computational evolution, VVHEVTL was the best solution for VP while AAP had multiple best solutions with the consensus sequence xxxxxRx (**Figure 7B**). This again lends support to the notion that the overlapping AAP heptapeptide region can be more flexible in the amino acid compositions for maintaining its function. In addition, the comparison between singly evolved and co-evolved QVKEVTQ/KSKRSRR-derived heptapeptide sequences provides an alternative means to investigate which amino acids are under overlap-evoked co-evolutionary constraints to what extent. Remarkable amino acid composition changes were observed at the P1, P3 and P7 in VP and P2 and P5 positions in AAP, indicating that these positions are under strong co-evolutionary constraints. Less pronounced changes were also observed in other positions except for the P2 and P5 in VP and P4 and P6 in AAP. Taken all together, our study using the experimental and computational evolution approaches demonstrates that, in the VP/AAP-overlapping heptapeptide region we studied, the two valine (V) residues in VP pose the primary structural and positional constraints and are the source of the co-evolutionary cascade, which subsequently restricts the choice of amino acids in AAP and those in other positions in VP directly and/or indirectly in concert with the AAP-originating biochemical constraints.

### K321 is Important for Viral Infectivity

**Figure 8:**
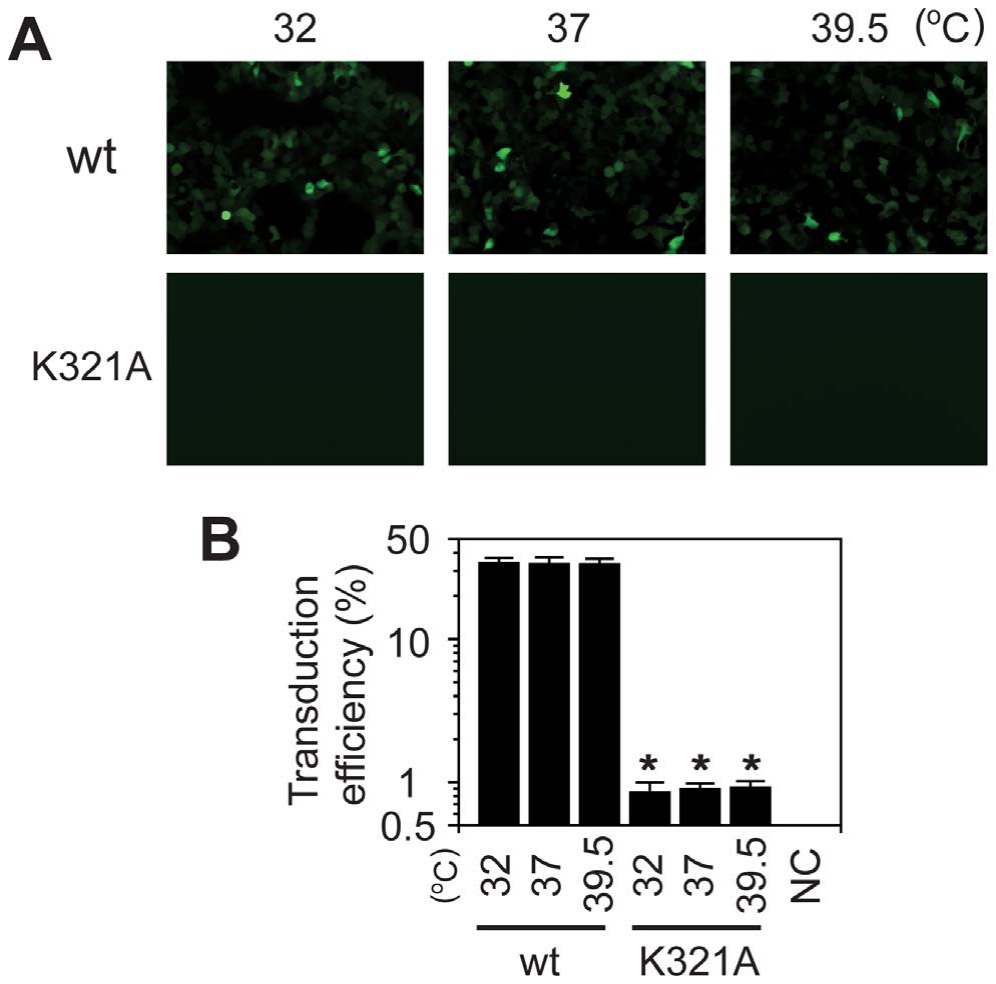
HEK293 cell transduction with wild type AAV2 or AAV2 K321A mutant expressing GFP. HEK293 cells were infected with double-stranded (ds) AAV2-CMV-GFP at MOI of 20,000 and maintained at 32, 37 or 39.5°C. Forty-eight hours post-infection, cells were observed under a fluorescent microscope (Panel A) and subjected to flow cytometry (Panel B). The experiment was done in quadruplicate. Asterisks indicate statistical significance with *P* = 0.000054 to 0.00032 (Student’s t-test) compared to the values of the corresponding wild type controls. Non-transduced cells were used as a negative control (NC).

The lysine (K) at P3 in VP (*i.e.*, K321) is strongly conserved in nature. However, to our surprise, lysine (K) was totally excluded from the amino acid position 321 in the experimental evolution and *in silico* evolution. We should note that our experimental directed evolution approach only considered whether or not VP3 mutants form viral particles and did not consider their infectivity. Therefore, we hypothesized that this lysine (K) plays an essential role in AAV infection. To address whether K321 is important for infectivity, we created AAV2 K321A mutant, and investigated its infectivity to HEK293 cells at 32, 37 and 39.5°C. As a result, we found that it displayed an infection-defective phenotype at all temperatures tested (**Figure 8**), demonstrating the essential functional role of lysine (K) at the position 321. A previous study by Wu et al. has shown that the K321A/E322A double alanine mutation makes infectious viral particles but results in a heat-sensitive phenotype, which infects cells at 32°C but not at 39.5°C [28]. Restoration of infectivity of K321A by introducing an additional mutation E322A [28], therefore indicates that the neutral nature of the K321/E322 positions is important not only for virion formation but also for infectivity.

### A Hypothetical Model of Co-evolution of the Evolutionarily Conserved QVKEVTQ/KSKRSRR Heptapeptide VP/AAP Motifs

**Figure 9:**
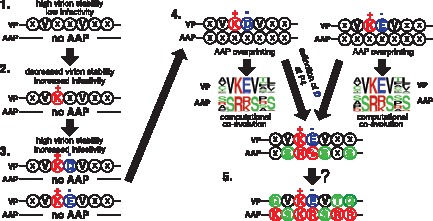
A hypothetical model for a mode of co-evolution of VP/AAP-overlapping heptapeptides. (1) The heptapeptide VP region in the evolutionarily most primitive form when no AAP exists is shown. (2) Acquisition of lysine (K) at VP P3 increases infectivity and is fixed preferentially, but may decrease virion stability. (3) Due to the neutral-neutral or basic-acidic structural constraints imposed on VP P3 and P4, aspartic acid (D) or glutamic acid (E) is favorably placed at P4. (4) AAP overprinting takes place on either xVKDVxx or xVKEVxx sequence motif of VP. A computational co-evolution analysis of xVKDVxx or xVKEVxx-coding DNA predicts evolutionary extinction of VP carrying aspartic acid (D) at P4 and emergence of AAP exhibiting a xSR(K/R)SxS consensus sequence. (5) The overlapping ORF further evolves into the present form by a mechanism that has yet to be elucidated.

With all this taken together, we propose a hypothetical model of the co-evolutionary mode of the heptapeptide VP/AAP motifs we focused on in this study (**Figure 9**). First, we need to point out that AAP ORF should be an ORF that has overprinted the VP ORF because AAP shows the following two features characteristic of overlapping ORFs: AAP is taxonomically restricted to only the genus *Dependovirus* [16], [17]; and AAP exhibits features of a disordered protein (**Figure S2**) [2], [5]. In our model, the primitive QVKEVTQ ORF in VP, which might have coded a heptapeptide that was different from the present-day sequence, but likely had the xVxxVxx sequence motif, was selected as the region where the primitive AAP would overprint a +1 frame-shifted ORF coding a heptapeptide with the AAP KSKRSRR function. Conforming to this assumption, back-translation of AAV2 VP1 into all possible nucleotide sequences and subsequent translation using the +1 frame reveals that the QVKEVTQ-coding nucleotide region is the best fit for accommodating the +1 frame-shifted heptapeptides showing the biochemical properties characteristic for those exhibiting the AAP KSKRSRR function (**Figure S3**). Once VP acquired lysine (K) at P3 (the position 321), lysine would become fixed at the position due to the improvement of infectivity, and this was presumably followed by acquisition of aspartic acid (D) or glutamic acid (E) at P4 due to the basic-acidic structural constraints imposed on VP P3 and P4 (**Figure 4**). Subsequently, overlap-evoked co-evolutionary constraints extinguished aspartic acid (D) and fixed glutamic acid (E) at P4 once AAP overprinting on VP took place. This hypothetical evolutionary process creates xVKEVxx/xSR(K/R)SxS motifs, which look quite similar to the sequence found in nature, QVKEVTQ/KSKRSRR.

## Discussion

We present here a new methodology that combines both experimental and computational approaches to understand the mode of co-evolution of viral overlapping ORFs and elucidate how individual amino acids in proteins coded by overlapping ORFs are structurally, functionally and co-evolutionarily constrained. Our original motive for the initiation of this study was to address a hypothesis that the evolutionarily highly conserved protein regions coded by the VP/AAP-overlapping ORFs, such as QVKEVTQ/KSKRSRR heptapeptide motifs we focused on in this study, would show significant evolutionary diversity if they were coded by independent ORFs. Since nature would not allow proteins coded by overlapping ORFs to evolve independently, conventional sequence alignment approaches are not able to address this hypothesis. Therefore, we established a new AAV directed evolution approach based on transcomplementation, where we let random VP and AAP mutants evolve into capsid-forming viable mutants by supplying AAP and VP expressed from separate DNA templates in trans, respectively, in HEK293 cells. By taking this experimental evolution approach in combination with Illumina sequencing, we could obtain a number of viable VP and AAP mutants showing significant sequence diversity. This demonstrates that the above-mentioned hypothesis is true at least to the heptapeptide motifs we chose for this study. When viewed in the light of gene therapy research, this observation may have implication in the development of new AAV vectors. This is because introduction of various mutations in the evolutionarily highly conserved N-terminal two-fifths of VP3 may potentially be tolerated and some of these new AAV species might have unforeseen attractive biological features for gene therapy applications. Further studies are needed to understand the degree of degeneracy of amino acid sequences in other regions in the VP/AAP-overlapping ORFs.

Various computational models have been utilized to study co-evolution of biological molecules. Computational methods for prediction of overlapping genes have been developed and successfully used to discover new viral overlapping genes [8], [10], [11]. Computational methods have also been used to identify co-evolved covariant amino acid residues in viral proteins and find structurally or functionary important residues that undergo co-evolution through intra- or inter-protein interactions [29], [30], [31]. These methods are all purely computational and bioinformatics-based methods. In contrast, the computational evolution method described here takes an approach distinct from the above-mentioned approaches in that it employs a data-driven computational model that empirically predicts how VP/AAP-overlapping ORFs co-evolve based on a large set of biological observations obtained from wet lab experiments. Such an approach was not realistic until recently, but has become possible through the emergence of the next-generation sequencing technologies that can generate a massive amount of data with minimal time and effort. The power of this data-driven computational approach is exemplified by our demonstration that the two serine (S) residues at P2 and P5 in the KSKRSRR motif in AAP are evolutionarily highly conserved not due to the structural or functional significance of these serine (S) residues in the context of AAP, but due primarily to the structural constraints originating from the superimposed QVKEVTQ motif in VP. The experimental evolution study showed that glycine (G), alanine (A) and proline (P) could substitute serine (S) residues to exert the AAP KSKRSRR function (**Figure 3C**). AAP has 5 serine (S)/threonine (T)-rich domains comprising 5 to 7 residues; and of these 5 S/T-rich domains, the two evolutionarily conserved ones located at T77 to T82 and S124 to T128 have recently been shown to tolerate amino acid substitutions in maintaining the capsid assembly function of AAP [18]. Based on the result of the mutagenesis study, Naumer et al. has suggested the functional redundancy of the S/T-rich motifs assuming that these motifs in fact have a functional role [18]. However, the results of our study raise an alternative possibility that the richness of serine (S) and threonine (T) residues in these two regions, T77 to T82 and S124 to T128, is a consequence of structural and/or functional constraints on the superimposed VP regions, which are also evolutionarily conserved. The combinatorial approach using experimental directed evolution and data-driven computational model presented here will answer this question when it is applied to the S/T-rich regions in our future experiments.

Although the new method is powerful in studying co-evolution of viral overlapping ORFs at individual amino acid levels, it should not be mistaken for a method that can perfectly reproduce how overlapping ORFs have evolved in nature. In nature, co-evolutionary constrains imposed on proteins coded by overlapping ORFs not only originate from the shared genetic information, but also may originate from intra- and inter-protein interactions of co-evolving amino acid residues. Examples of the latter are covariance of viral envelope amino acid residues that are non-contiguous in primary structure [30], [32], [33] and co-evolution of ligands and their cognate receptors [34]. In addition, virus-host interaction may play a role in viral evolution, where viruses have evolved a plethora of strategies to counteract host innate and acquired immune responses that eliminate pathogens. Although AAV does not express any known modulators for immune evasion, low immunogenicity of AAV may be a trait acquired by evolution. In the method presented here, we applied a simplified system in which we let only the heptapeptides of interest undergo evolution and did not allow other regions or proteins to evolve during the experimental period or did not consider evolutionary adaptation processes in the context of virus-host interactions. Therefore, the molecular evolution we simulated in cultured cells and *in silico* is an artificial evolutionary process and does not reproduce all aspects of evolution in nature. Although this caveat needs to be kept in mind, the new method should be valid for studying conserved amino acids in proteins coded by overlapping ORFs within the AAV genome because this artificial evolution system could successfully interpret the peculiar sequence features of the heptapeptide motifs incorporated into the context of the present-day amino acid sequences of VP and AAP. The simplest structure of AAV and lack of expression of viral immune modulators would make AAV a straightforward model to study co-evolution of viral overlapping ORFs using the method reported here.

In summary, the new method successfully dissects the mode of co-evolution of overlapping ORFs at individual amino acid levels and significantly advances our understanding of how viral overlapping genes emerged and have undergone co-evolution. This methodology can be applied to study of other regions in the overlapping AAV genes, will potentially be extendable to study of overlapping ORFs in other viral species, and will allow us to address questions that are difficult to answer by conventional sequence alignment-based approaches.

## Materials and Methods

### Plasmid Construction

pCMV-FLAG-cmAAP is a plasmid expressing a codon-modified version of AAV2 AAP with a FLAG tag under the control of the human cytomegalovirus (CMV) immediately early gene enhancer/promoter. Homology between the cmAAP ORF and native sequence was 61.9%. pAAV2-RepVP3 is a plasmid that expresses all the Rep proteins, VP3 protein, but does not express VP1, VP2 or AAP. pAAV2-RepVP3-Lib-BB is the backbone plasmid for the creation of pAAV2-RepVP3-Lib-0. pAAV2-RepVP3-Lib-0 is the plasmid library with which we produced AAV2-RepVP3-Lib-1 virus library carrying random heptapeptides in place of QVKEVTQ in the VP3 protein. pAAV2-RepVP3-Lib-0 expresses all the Rep proteins, mutant VP3, but does not express VP1, VP2 or AAP. pAAV2-CMV-cmAAP is a plasmid with which we produced an AAV2 vector expressing cmAAP. pAAV2-CMV-cmAAP-Lib-BB is the backbone plasmid for the creation of pAAV2-CMV-cmAAP-Lib-0. pAAV2-CMV-cmAAP-Lib-0 is the plasmid library with which we produced the AAV2-CMV-cmAAP-Lib-1 virus library carrying random heptapeptides in place of KSKRSRR in the AAP protein. Please refer to **Text S1** for complete descriptions of the plasmids used in the study.

### Cells

Human embryonic kidney (HEK) 293 cells, AAV293, were purchased from Stratagene. HEK293 cells were grown in Dulbecco’s modified Eagle’s medium (DMEM, Lonza, Basel, Switzerland) supplemented with 10% fetal bovine serum (FBS), L-glutamine, and penicillin-streptomycin.

### AAV Production

AAV VP1/VP2/VP3 particles (*i.e.*, fully infectious particles if they are wild type) and non-infectious VP3 only particles were produced in HEK293 cells by an adenovirus-free plasmid transfection method as previously described [35]. The plasmids used for the production of each viral preparation are summarized in **Table S4**. The VP1/VP2/VP3 AAV particles used for cell infection assays were purified by 2 cycles of cesium chloride ultracentrifugation [36] unless otherwise noted. DNase I-resistant AAV particle titers were determined by a quantitative dot blot assay.

### Experimental Directed Evolution and Illumina Sequencing

AAV2-RepVP3-Lib-1 and AAV2-CMV-cmAAP-Lib-1 virus libraries were produced in HEK293 cells by the adenovirus-free plasmid transfection method using pAAV2-RepVP3-Lib-0 and pAAV2-CMV-cmAAP-Lib-0 plasmids, respectively. To minimize the phenotype-genotype dissociation problem in the libraries, we applied the modified 1-step method we have reported previously [22]. Forty-eight hours post-transfection of the plasmid DNA, cells were harvested, and the AAV2-RepVP3-Lib-1 and AAV2-CMV-cmAAP-Lib-1 viral particles were released from the cells by three cycles of freezing and thawing. Nuclease-resistant viral DNA was recovered from the crude cell lysates and used as a PCR template to amplify the random 21 nucleotide-long region. The PCR-amplified fragments were cloned into either pAAV2-RepVP3-Lib-BB or pAAV2-CMV-cmAAP-Lib-BB and the second round plasmid libraries, pAAV2-RepVP3-Lib-1 and pAAV2-CMV-cmAAP-Lib-1, were created. This cycle was repeated three times until we obtained AAV2-RepVP3-Lib-3 and AAV2-CMV-cmAAP-Lib-3 viral particles. At each round of selection (i.e., Lib-0, Lib-1, Lib-2 and Lib-3. Please refer to **Figure 2B**), the random 21 nucleotide-long regions of the AAV genome were amplified by PCR with sample DNA barcode-indexed primers (**Table S5**) and characterized by multiplexed Illumina sequencing [37] using HiSeq 2000 (**Figure 2C**). Please refer to **Text S1** for complete description of the directed evolution procedure and Illumina sequencing.

### Cell Culture Experiments

Capability of viral capsid formation of mutant VP3 protein and the capsid assembly activating function of mutant AAP proteins were assessed by analyzing the virus production yield of mutant AAV2-RepVP3 and mutant AAV2-CMV-cmAAP, respectively, in HEK293 cells. Virus infectivity of the capsid mutants was assessed with HEK293 cells using double-stranded (ds) AAV2-CMV-GFP vector coated with corresponding mutant VP proteins. Please refer to **Text S1** for complete description of the cell culture experiments.

### Computational Directed Evolution

An evolutionary algorithm was developed to computationally evolve given nucleotide sequences into nucleotides that encode heptapeptides exhibiting biochemical properties and amino acid compositions similar to those of the heptapeptides we identified experimentally. To this end, we defined the fitness function in the evolutionary algorithm as the sum of the objective function term and the penalty function term that comprise the sum of the sub-objective functions and the sub-penalty functions, respectively. Regarding the sub-objective functions, the information about the biochemical properties and amino acid compositions of each of the VP/AAP-overlapping heptapeptide target regions (**Tables S6** and **S7**) was transformed into 4 sub-objective functions (molecular weight (MW), isoelectric point (IP), GRAVY and amino acid composition (AA)) for each of VP and AAP. The present study has demonstrated that MW, IP, GRAVY and AA are the properties important for viability, providing the rationale for having these 4 sub-objective functions in the fitness function. As for the sub-penalty functions, we defined 3 sub-penalty functions for MW, IP and GRAVY, for each of VP and AAP, based on the cut-off values described in **Table S8**. For VP, we had additional 5 sub-penalty functions associated with strong molecular weight and hydropathic constraints imposed on P1, P2 and P3 (**Figure S4**) and with the electrostatic constraints imposed on P3 and P4 (**Figure 4**). In the computational evolution procedure, we created the initial population consisting of 200 individuals from a single ancestral DNA by introducing one random nucleotide substitution, and evolved them using a linear ranking selection scheme [38] for 200 generations in a way that minimizes the fitness function. We evolved 200 populations in parallel and collected information about the biochemical properties and amino acid compositions of the VP and AAP heptapeptides from all the 40,000 individuals in the 200th generation. Please refer to **Text S1** for complete description of the algorithm used for the computational evolution.

### Bioinformatics

Nucleotide sequence information of 128 AAV species was collected from GenBank. Conservation scores were determined by ConSurf with the default parameters [39]. Structural disorder of the VP and AAP proteins within the VP/AAP-overlapping ORFs was analyzed using PONDR VSL1 predictor [40]. Secondary structure classes (*i.e.*, α-helix, β-strand and coil) of the VP and AAP heptapeptides were predicted using the Discrimination of Secondary Structure Class (DSC) algorithm [27]. GenBank accession numbers for the 128 AAV species are found in **Text S1**.

### Statistical Analysis

Differences in AAV viral particle production yields and AAV infection or transduction efficiencies were statistically assessed by the two-tailed Student’s t-test. A binomial test was used to statistically assess the probability of the absence of stop codons in heptapeptides in a given peptide population. The contents of amino acids in ordered and disordered states in the VP and AAP proteins within the VP/AAP-overlapping region were statistically compared using a chi-square test. In other statistical analyses in which two datasets were compared, the two-tailed Mann-Whitney U-test was used. A power analysis was performed in comparisons of chemical properties of heptapeptides between a viable mutant group and the pool of random peptides in the original library as detailed in each comparison in the text.

### NCBI Sequence Accession Numbers

Raw Illumina sequencing data have been submitted to National Center for Biotechnology Information (NCBI) Sequence Read Archive (SRA) and can be accessed under accession numbers SRP021031 and SRP021053.

## Supporting Information

Figure S1 **Prediction of secondary structures of the VP and AAP heptapeptide regions.** Prediction results obtained by the DSC method [27] are indicated as stacked bars. The height of each bar in the stack indicates the percentage of heptapeptides that are predicted to be in the secondary structure in each class. Amino acid positions in the heptapeptides are indicated as P1, P2 and so on, and the type of the heptapeptides (*i.e.*, VP heptapeptides or AAP heptapeptides) is shown above each panel. **(A** and **E)** The 930372 random VP heptapeptides and 554743 random AAP heptapeptides in the libraries Lib-0. **(B** and **F)** The experimentally evolved 143 viable VP heptapeptides and 492 viable AAP heptapeptides in the libraries Lib-3. **(C** and **G)** The 40000 VP heptapeptides and 40000 AAP heptapeptides singly evolved from random nucleotide sequences *in silico* under no co-evolutionary constraints. **(D** and **H)** The 40000 VP heptapeptides and 40000 AAP heptapeptides co-evolved from random nucleotide sequences *in silico*. **(I, J, K, L, M** and **N)** Computer-generated 10000 random VP heptapeptides. Twenty different amino acids are randomly assigned to the positions indicated with “X”. In panels J, K, L, M and N, a specific amino acid (*i.e.*, valine (V), isoleucine (I) or aspartic acid (N)) is assigned to P2 or P5 as indicated. **(O)** Experimentally evolved 21 viable VP heptapeptides that have amino acid residues other than valine (V) (*i.e.*, non-valine or NV) at P2 and P5. **(P)** 217 VP heptapeptides representing 61 different populations that singly evolved from random nucleotide sequences *in silico* and have amino acid residues other than valine (V) (*i.e.*, non-valine or NV) at P2 and P5.
(EPS)

Figure S2 **PONDR predictor values of the VP and AAP proteins within the VP/AAP-overlapping ORFs in the AAV2 genome.** The amino acid sequences of the VP and AAP proteins of 204 amino acids in length were subjected to the PONDR analysis to predict disordered protein regions [40]. Regions showing values of 0.5 or more are predicted to be disordered. The analysis reveals that AAP contains more amino acids in a disordered state than VP (chi-square value = 108.71, degree of freedom = 1, *P* = 1.87×10^−25^).
(EPS)

Figure S3 **Fitness landscape for the AAP KSKRSRR motif function in the AAV** ***cap*** **gene.** Using a sliding window of 8 amino acids, the AAV2 VP1 protein of 735 amino acids was back-translated into all possible nucleotide sequences, which were subsequently translated into all possible heptapeptides with no stops using the frame-shifted +1 ORF. A total of 9877978 heptapeptides with no stops could be translated at a various number per window (0 to 89244 per window), and their local fitness was assessed by their isoelectric points (Panel A) and fitness function scores (Panel B). **(A)** Percentage of heptapeptides exhibiting isoelectric point (IP) ≥10.48 among all possible +1 ORF heptapeptides in each window. 10.48 was the lowest of the IP values of all possible QVKEVTQN-derived +1 ORF heptapeptides. The highest unique peak was observed at the positions corresponding to 319-QVKEVTQN, 320-VKEVTQND and 321-KEVTQNDG windows (black lines indicated with an arrowhead). **(B)** Means of AAP fitness function (*f_aap(x)* ) scores of all possible +1 ORF heptapeptides in each window. Please note that the lower the score is, the better the fitness is. Scores in the above three windows were among the lowest (black lines indicated with an arrowhead) and formed a deep valley. Due to the presence of a stop codon(s) within the +1 ORF heptapeptides, the following windows have no values; the windows starting from amino acid positions 427 to 433 and 551 to 557.
(EPS)

Figure S4 **Chemical properties of the 143 viable VP heptapeptide mutants analyzed with a sliding window of two amino acids.** **(A)** Molecular weight (MW); **(B)** Isoelectric point (IP); and **(C)** GRAVY score. Upper and lower bands indicate histograms of the viable 143 VP heptapeptide mutants found in Lib-3 and random heptapeptide mutants found in Lib-0, respectively.
(EPS)

Table S1 The amino acid compositions (%) in the viable 143 VP and 492 AAP heptapeptide mutants.
(DOCX)

Table S2 The amino acid compositions (%) in the original VP and AAP heptapeptide libraries (*i.e.*, Lib-0).
(DOCX)

Table S3 Statistical comparison of amino acid compositions between top-ranked heptapeptides in Lib-3 and random heptapeptides in Lib-0.
(DOCX)

Table S4 AAV and helper plasmids used for AAV production.
(DOCX)

Table S5 Sample DNA barcode-indexed PCR primers for Illumina sequencing.
(DOCX)

Table S6 Means and standard deviations of MW, IP and GRAVY scores of the viable VP and AAP heptapeptides.
(DOCX)

Table S7 VP and AAP amino acid matrices used for the evolutionary algorithm.
(DOCX)

Table S8 Cut-off values for sub-penalty functions.
(DOCX)

Text S1 **Supporting Materials and Methods.**
(DOCX)
