# Supplementary material for: An Experimental and Computational Evolution-Based Method to Study a Mode of Co-evolution of Overlapping Open Reading Frames in the AAV2 Viral Genome
Source: PLoS One. 2013 Jun 24;8(6):e66211. doi: 10.1371/journal.pone.0066211 (PMC3691236; doi:10.1371/journal.pone.0066211)
Supplement: Table S1 — The amino acid compositions (%) in the viable 143 VP and 492 AAP heptapeptide mutants. (DOCX) [file pone.0066211.s005.docx]

|  | VP (143^b^) | | | | | | |  | AAP (492^b^) | | | | | | |
| --- | --- | --- | --- | --- | --- | --- | --- | --- | --- | --- | --- | --- | --- | --- | --- |
|  |  | Amino acid position | | | | |  |  |  | Amino acid position | | | | |  |
| Amino acid | P1  (Q)^a^ | P2  (V) | P3  (K) | P4  (E) | P5  (V) | P6  (T) | P7  (Q) |  | P1  (K) | P2  (S) | P3  (K) | P4  (R) | P5  (S) | P6  (R) | P7  (R) |
| A | 14.0 | 4.2 | 0.0 | 3.5 | 7.0 | 5.6 | 0.7 |  | 16.1 | 8.7 | 7.9 | 10.8 | 5.1 | 6.9 | 11.4 |
| C | 19.6 | 3.5 | 2.8 | 8.4 | 5.6 | 13.3 | 11.2 |  | 0.6 | 0.4 | 1.6 | 0.8 | 1.2 | 0.4 | 1.8 |
| D | 0.0 | 0.0 | 0.0 | 10.5 | 0.0 | 0.7 | 2.1 |  | 0.2 | 0.4 | 0.4 | 0.2 | 0.4 | 0.0 | 0.2 |
| E | 0.7 | 0.0 | 0.0 | 2.8 | 3.5 | 0.7 | 1.4 |  | 2.0 | 0.8 | 1.0 | 1.6 | 1.8 | 1.2 | 0.2 |
| F | 0.0 | 0.0 | 19.6 | 12.6 | 1.4 | 4.9 | 5.6 |  | 0.2 | 0.0 | 0.0 | 0.4 | 0.0 | 0.2 | 0.4 |
| G | 18.2 | 2.1 | 0.0 | 2.8 | 0.7 | 19.6 | 2.1 |  | 25.2 | 34.8 | 26.6 | 21.5 | 18.5 | 25.8 | 40.7 |
| H | 0.0 | 0.0 | 23.8 | 1.4 | 0.0 | 2.8 | 4.9 |  | 1.0 | 0.4 | 1.8 | 0.8 | 0.8 | 0.8 | 0.8 |
| I | 0.0 | 4.9 | 0.0 | 2.8 | 1.4 | 0.7 | 1.4 |  | 0.0 | 0.0 | 0.0 | 0.2 | 0.0 | 0.0 | 0.0 |
| K | 0.7 | 0.0 | 0.0 | 0.0 | 0.0 | 0.7 | 0.0 |  | 0.2 | 1.4 | 1.6 | 2.0 | 2.4 | 1.8 | 0.2 |
| L | 0.7 | 5.6 | 6.3 | 2.1 | 2.1 | 6.3 | 26.6 |  | 1.6 | 1.0 | 2.0 | 1.4 | 2.2 | 2.2 | 1.2 |
| M | 4.2 | 3.5 | 8.4 | 2.1 | 0.0 | 1.4 | 3.5 |  | 0.4 | 0.6 | 0.6 | 0.6 | 0.4 | 0.6 | 0.4 |
| N | 1.4 | 0.7 | 0.0 | 2.8 | 0.7 | 6.3 | 0.7 |  | 0.0 | 0.2 | 0.0 | 0.0 | 0.4 | 0.6 | 0.4 |
| P | 0.0 | 1.4 | 0.7 | 0.0 | 2.8 | 0.0 | 0.0 |  | 13.0 | 11.6 | 6.9 | 12.0 | 6.5 | 4.5 | 9.3 |
| Q | 2.1 | 0.0 | 0.7 | 0.0 | 2.1 | 0.7 | 2.8 |  | 2.8 | 0.8 | 1.4 | 2.0 | 2.0 | 1.8 | 0.6 |
| R | 0.7 | 0.0 | 6.3 | 0.0 | 0.0 | 2.1 | 0.0 |  | 27.0 | 27.8 | 35.2 | 26.4 | 46.5 | 40.9 | 20.1 |
| S | 11.9 | 2.8 | 0.0 | 13.3 | 11.2 | 19.6 | 4.2 |  | 1.6 | 2.6 | 4.5 | 6.5 | 5.1 | 3.3 | 4.1 |
| T | 4.9 | 2.8 | 0.0 | 8.4 | 4.9 | 7.7 | 0.0 |  | 2.2 | 2.6 | 2.0 | 4.5 | 1.6 | 3.0 | 3.3 |
| V | 21.0 | 68.5 | 0.0 | 14.0 | 56.6 | 3.5 | 24.5 |  | 3.9 | 4.1 | 4.5 | 5.5 | 2.2 | 3.3 | 3.5 |
| W | 0.0 | 0.0 | 25.2 | 12.6 | 0.0 | 0.0 | 2.8 |  | 1.8 | 1.4 | 1.4 | 2.2 | 2.6 | 2.2 | 0.6 |
| Y | 0.0 | 0.0 | 6.3 | 0.0 | 0.0 | 3.5 | 5.6 |  | 0.0 | 0.2 | 0.4 | 0.4 | 0.0 | 0.4 | 0.8 |

**Table S1. Amino acid compositions (%) in the viable 143 VP and 492 AAP heptapeptide mutants.**

^a^The amino acids in parentheses are those found in the wild type VP and AAP proteins.

^b^The number of heptapeptides analyzed.
