# Supplementary material for: An Experimental and Computational Evolution-Based Method to Study a Mode of Co-evolution of Overlapping Open Reading Frames in the AAV2 Viral Genome
Source: PLoS One. 2013 Jun 24;8(6):e66211. doi: 10.1371/journal.pone.0066211 (PMC3691236; doi:10.1371/journal.pone.0066211)
Supplement: Table S2 — The amino acid compositions (%) in the original VP and AAP heptapeptide libraries (i.e., Lib-0). (DOCX) [file pone.0066211.s006.docx]

|  | VP (930372^b^) | | | | | | |  | AAP (554743^b^) | | | | | | |
| --- | --- | --- | --- | --- | --- | --- | --- | --- | --- | --- | --- | --- | --- | --- | --- |
|  |  | Amino acid position | | | | |  |  |  | Amino acid position | | | | |  |
| Amino acid | P1  (Q)^a^ | P2  (V) | P3  (K) | P4  (E) | P5  (V) | P6  (T) | P7  (Q) |  | P1  (K) | P2  (S) | P3  (K) | P4  (R) | P5  (S) | P6  (R) | P7  (R) |
| A | 5.7 | 6.9 | 7 | 5.5 | 4.4 | 4.8 | 4.2 |  | 15.9 | 11.8 | 8.3 | 11.9 | 6.6 | 8.9 | 12.8 |
| C | 4.3 | 3.8 | 5.7 | 4.9 | 3.7 | 3.9 | 4.5 |  | 1.4 | 1.7 | 3.6 | 2.3 | 2.7 | 2.3 | 3 |
| D | 2.5 | 2.9 | 2.2 | 2.9 | 2.7 | 2.5 | 2.7 |  | 0.9 | 0.8 | 1.6 | 1.4 | 1.2 | 1.8 | 1.8 |
| E | 4 | 3.3 | 2.4 | 3.9 | 3.2 | 3.9 | 6 |  | 2.8 | 2.9 | 2.3 | 3.4 | 2.7 | 3.7 | 3.1 |
| F | 1.4 | 2.8 | 3.6 | 5.3 | 3.8 | 5.2 | 6.5 |  | 0.4 | 0.4 | 0.9 | 1.1 | 1.4 | 1.4 | 1.4 |
| G | 17.8 | 17.6 | 13.2 | 14.4 | 13 | 17.8 | 13.8 |  | 27.1 | 32.9 | 27.4 | 20.5 | 19.4 | 22.9 | 26.3 |
| H | 2 | 2.2 | 1.7 | 2.1 | 2.2 | 1.9 | 2.4 |  | 0.5 | 0.5 | 1.3 | 1.2 | 1.7 | 1.3 | 1 |
| I | 1.9 | 1.8 | 2.1 | 2.3 | 2.1 | 1.9 | 1.8 |  | 0.2 | 0.2 | 0.5 | 0.6 | 0.7 | 0.9 | 0.7 |
| K | 3.3 | 3 | 3 | 5.2 | 4.8 | 3 | 6.1 |  | 0.5 | 0.8 | 0.8 | 1.6 | 1.4 | 2 | 1.3 |
| L | 7.8 | 6.9 | 6.4 | 7 | 10 | 11 | 8.1 |  | 2.9 | 2.7 | 4 | 4.9 | 7.6 | 5.4 | 4.1 |
| M | 3.1 | 2.3 | 2.7 | 2.1 | 2.8 | 2.4 | 1.9 |  | 0.5 | 0.6 | 0.7 | 1.2 | 1.3 | 1.7 | 1.1 |
| N | 2.4 | 2 | 1.9 | 3.5 | 4.5 | 2.1 | 2.5 |  | 0.2 | 0.2 | 0.6 | 0.8 | 0.7 | 1.1 | 1 |
| P | 3 | 4 | 4.9 | 3.7 | 3.2 | 3.1 | 3.5 |  | 11.5 | 8.9 | 7.9 | 10.9 | 7.4 | 6.9 | 6.8 |
| Q | 2.6 | 2.6 | 1.6 | 2.2 | 2.2 | 2.3 | 4.5 |  | 1.9 | 1.8 | 1.7 | 2.8 | 3.7 | 2.8 | 1.6 |
| R | 9.7 | 10.1 | 12.1 | 8.9 | 9.4 | 7.8 | 6.7 |  | 15.8 | 16.3 | 17.4 | 12.3 | 20 | 14.6 | 10.2 |
| S | 5.6 | 5.5 | 8 | 7 | 7.8 | 5.4 | 5.6 |  | 3.6 | 3.5 | 5.2 | 6 | 5.1 | 5.2 | 6.1 |
| T | 4.2 | 4 | 7 | 4.2 | 3.8 | 2.8 | 2.7 |  | 2.8 | 2.2 | 3 | 4.6 | 3 | 3.9 | 4.2 |
| V | 10.2 | 12 | 8.2 | 8.4 | 8.2 | 10.1 | 8.4 |  | 6.7 | 6.5 | 6.3 | 6.7 | 5.9 | 7.4 | 7.7 |
| W | 6.9 | 4.4 | 4.4 | 4 | 5.6 | 6.1 | 5.1 |  | 4.2 | 4.8 | 5.7 | 5.2 | 6.7 | 4.8 | 5 |
| Y | 1.7 | 1.9 | 1.9 | 2.6 | 2.4 | 2.1 | 3 |  | 0.2 | 0.2 | 0.6 | 0.7 | 0.8 | 1 | 0.9 |

**Table S2. Amino acid compositions (%) in the original VP and AAP heptapeptide libraries (*i.e.*, Lib-0).**

^a^The amino acids in parentheses are those found in the wild type VP and AAP proteins.

^b^The number of heptapeptides analyzed
