# Supplementary material for: An Experimental and Computational Evolution-Based Method to Study a Mode of Co-evolution of Overlapping Open Reading Frames in the AAV2 Viral Genome
Source: PLoS One. 2013 Jun 24;8(6):e66211. doi: 10.1371/journal.pone.0066211 (PMC3691236; doi:10.1371/journal.pone.0066211)
Supplement: Table S3 — Statistical comparison of amino acid compositions between top-ranked heptapeptides in Lib-3 and random heptapeptides in Lib-0. (DOCX) [file pone.0066211.s007.docx]

**Table S3. Statistical comparison of amino acid compositions between top-ranked heptapeptides in Lib-3 and random heptapeptides in Lib-0.**

| Ranking in Lib-3 | *P* value, Mann-Whitney U-test | |
| --- | --- | --- |
|  | VP heptapeptide | AAP heptapeptide |
| 1-50 | 0.000011 | 0.000011 |
| 51-100 | 0.000011 | 0.000011 |
| 101-150 | 0.000011 | 0.000011 |
| 151-200 | 0.000011 | 0.000011 |
| 201-250 | 0.000011 | 0.000487 |
| 251-300 | n.d. | 0.000011 |
| 301-350 | n.d. | 0.000011 |
| 351-400 | n.d. | 0.001050 |
| 401-450 | n.d. | 0.000011 |
| 451-500 | n.d. | 0.049860 |
| 501-550 | n.d. | 0.000487 |
| lowest-ranked^a^ | 0.984975 | 0.393048 |

^a^Lowest-ranked peptides are those that were counted only once in the Illumina sequencing analysis of Lib-3 libraries. There were 57901 and 14412 lowest-ranked peptides among the 67565 and 22911 peptides in the VP and AAP Lib-3 libraries.

n.d., not done.

Statistical significance of the difference in amino acid compositions between two peptide populations was assessed using Mann-Whitney U-test in the following manner. First, amino acid compositions of 50 heptapeptides were determined in each group. For the lowest-ranked peptides, 10 sets of 50 heptapeptides were generated in such a way that 50 peptides were randomly selected from each of the lowest-ranked peptide pools to make a set of 50 peptides. Likewise, 10 sets of 50 peptides were selected randomly from the original libraries to create 10 sets of reference controls. Second, we compared amino acid compositions between each of the groups containing top-ranked positively selected peptides and non-selected reference controls, and calculated statistical variance (*Vsc*) using the following formula:

*Vsc = Σ (Sij-Cij)^2^ / N, i ∈ {1, 2, 3, 4, 5, 6, 7}, j∈{A, C, D, E, F, G, H, I, K, L, M, N, P, Q, R, S, T, V, W, Y}*

In this formula, *i* and *j* represent the amino acid position in the heptapeptide and amino acids, respectively. *Sij* and *Cij* are the frequencies at which amino acid *j* is found at the position *i* (for example, please refer to **Table S1**) in a positively-selected group and a reference control group, respectively. *Σ (Sij-Rij)^2^*  is the sum of *(Sij-Rij)^2^*  for all the *ij* combinations. *N* is the number of the *ij* combinations (*i.e.*, *N* = 140). A total of 10 *Vsc* values were calculated for each group by comparing the group and 10 reference control groups. We also obtained a total of 10 *Vcc* values, which represent statistical variance between randomly selected 2 reference control sets, in the same manner as that for *Vsc*. Then the obtained *Vsc* and *Vcc* values were compared by the two-tailed Mann-Whitney U-test to investigate whether there is a significant difference in amino acid compositions between the two groups.
