# Supplementary material for: An Experimental and Computational Evolution-Based Method to Study a Mode of Co-evolution of Overlapping Open Reading Frames in the AAV2 Viral Genome
Source: PLoS One. 2013 Jun 24;8(6):e66211. doi: 10.1371/journal.pone.0066211 (PMC3691236; doi:10.1371/journal.pone.0066211)
Supplement: Table S4 — AAV and helper plasmids used for AAV production. (DOCX) [file pone.0066211.s008.docx]

| Virus name | Plasmid function | Plasmid name |
| --- | --- | --- |
| AAV2-RepVP3-Lib (VP3 only particles) | AAV plasmid | pAAV2-RepVP3-Lib |
|  | Adenovirus helper plasmid | pHelper |
|  | AAP helper plasmid | pCMV-FLAG-cmAAP |
|  |  |  |
| AAV2-CMV-cmAAP-Lib (VP3 only particles) | AAV plasmid | pAAV2-CMV-cmAAP-Lib |
|  | AAV helper plasmid | pHLP-RepVP3 |
|  | Adenovirus helper plasmid | pHelper |
|  |  |  |
| AAV2-RepVP3 wt or mt (VP3 only particles) | AAV plasmid | pAAV2-RepVP3 wt or mt |
|  | Adenovirus helper plasmid | pHelper |
|  | AAP helper plasmid | pCMV-FLAG-cmAAP |
|  |  |  |
| AAV2-CMV-cmAAPwt or mt (VP3 only particles) | AAV plasmid | pAAV2-CMV-cmAAPwt or mt |
|  | Adenovirus helper plasmid | pHelper |
|  | AAV helper plasmid | pHLP-RepVP3 |
|  |  |  |
| dsAAV2-CMV-GFP, wild type or VP mutants (VP1+VP2+VP3 particles) | AAV plasmid | pEMBL-CMV-GFP |
|  | AAV helper plasmid | pHLP22 with or without a VP mutation |
|  | Adenovirus helper plasmid | pHelper |
|  | AAP helper plasmid | pCMV-FLAG-cmAAP |

**Table S4. AAV and helper plasmids used for AAV production.**
