# Supplementary material for: An Experimental and Computational Evolution-Based Method to Study a Mode of Co-evolution of Overlapping Open Reading Frames in the AAV2 Viral Genome
Source: PLoS One. 2013 Jun 24;8(6):e66211. doi: 10.1371/journal.pone.0066211 (PMC3691236; doi:10.1371/journal.pone.0066211)
Supplement: Table S5 — Sample DNA barcode-indexed PCR primers for Illumina sequencing. (DOCX) [file pone.0066211.s009.docx]

| **Table S5. Sample DNA barcode-indexed PCR primers for Illumina sequencing.** | |
| --- | --- |
| Primer name | Primer sequence^a^ |
| T07-23 QVKEVTQ Forward-1 | 5' **TGG**ACTTCAAGCTTTTCAACATC 3' |
| T07-23 QVKEVTQ Forward-2 | 5' G**GGC**ACTTCAAGCTTTTCAACATC 3' |
| T07-23 QVKEVTQ Forward-3 | 5' AA**CTC**ACTTCAAGCTTTTCAACATC 3' |
| T07-23 QVKEVTQ Forward-4 | 5' CGT**AAT**ACTTCAAGCTTTTCAACATC 3' |
| T07-23 QVKEVTQ Forward-5 | 5' TTGG**TCC**ACTTCAAGCTTTTCAACATC 3' |
| T07-23 QVKEVTQ Forward-6 | 5' **GCT**ACTTCAAGCTTTTCAACATC 3' |
| T07-23 QVKEVTQ Forward-7 | 5' A**CAA**ACTTCAAGCTTTTCAACATC 3' |
| T07-23 QVKEVTQ Forward-8 | 5' CGAC**AGA**ACTTCAAGCTTTTCAACATC 3' |
| T07-23 QVKEVTQ Forward-9 | 5' **TTT**ACTTCAAGCTTTTCAACATC 3' |
| T07-23 QVKEVTQ Reverse-1 | 5' CG**TGG**ATCGTCGTCGTACCGTCATT 3' |
| T07-33 QVKEVTQ Reverse-2 | 5' GAG**GGC**ATCGTCGTCGTACCGTCATT 3' |
| T07-33 QVKEVTQ Reverse-3 | 5' TTCT**CTC**ATCGTCGTCGTACCGTCATT 3' |
| T07-33 QVKEVTQ Reverse-4 | 5' **AAT**ATCGTCGTCGTACCGTCATT 3' |
| T07-33 QVKEVTQ Reverse-5 | 5' G**TCC**ATCGTCGTCGTACCGTCATT 3' |
| T07-33 QVKEVTQ Reverse-6 | 5' TT**GCT**ATCGTCGTCGTACCGTCATT 3' |
| T07-33 QVKEVTQ Reverse-7 | 5' AAC**CAA**ATCGTCGTCGTACCGTCATT 3' |
| T07-33 QVKEVTQ Reverse-8 | 5' T**AGA**ATCGTCGTCGTACCGTCATT 3' |
| T07-33 QVKEVTQ Reverse-9 | 5' CC**TTT**ATCGTCGTCGTACCGTCATT 3' |
| T08-34 KSKRSRR Forward-1 | 5' **ATTG**CTAGTTCAAGCCTGACCTTC 3' |
| T08-34 KSKRSRR Forward-2 | 5' G**ACGT**CTAGTTCAAGCCTGACCTTC 3' |
| T08-34 KSKRSRR Forward-3 | 5' AA**GCAG**CTAGTTCAAGCCTGACCTTC 3' |
| T08-34 KSKRSRR Forward-4 | 5' CGT**CGGG**CTAGTTCAAGCCTGACCTTC 3' |
| T08-34 KSKRSRR Forward-5 | 5' TTGG**GACG**CTAGTTCAAGCCTGACCTTC 3' |
| T08-34 KSKRSRR Forward-6 | 5' **CTGA**CTAGTTCAAGCCTGACCTTC 3' |
| T08-34 KSKRSRR Forward-7 | 5' A**ACTA**CTAGTTCAAGCCTGACCTTC 3' |
| T08-34 KSKRSRR Forward-8 | 5' CGAC**GGCA**CTAGTTCAAGCCTGACCTTC 3' |
| T08-34 KSKRSRR Forward-9 | 5' **GTCT**CTAGTTCAAGCCTGACCTTC 3' |
| T08-34 KSKRSRR Forward-10 | 5' A**TGTT**CTAGTTCAAGCCTGACCTTC 3' |
| T08-34 KSKRSRR Forward-11 | 5' TG**TCTC**CTAGTTCAAGCCTGACCTTC 3' |
| T08-34 KSKRSRR Forward-12 | 5' CAA**TTTA**CTAGTTCAAGCCTGACCTTC 3' |
| T08-34 KSKRSRR Forward-13 | 5' GTGG**CTAC**CTAGTTCAAGCCTGACCTTC 3' |
| T08-34 KSKRSRR Forward-14 | 5' **CGTA**CTAGTTCAAGCCTGACCTTC 3' |
| T08-34 KSKRSRR Forward-15 | 5' TGC**ATGC**CTAGTTCAAGCCTGACCTTC 3' |
| T08-34 KSKRSRR Forward-16 | 5' GAAC**TTAG**CTAGTTCAAGCCTGACCTTC 3' |
| T08-34 KSKRSRR Forward-17 | 5' **AAAC**CTAGTTCAAGCCTGACCTTC 3' |
| T08-34 KSKRSRR Reverse-1 | 5' CG**ATTG**AGTCTTCTCCGGACAGTCAT 3' |
| T08-34 KSKRSRR Reverse-2 | 5' GAG**ACGT**AGTCTTCTCCGGACAGTCAT 3' |
| T08-34 KSKRSRR Reverse-3 | 5' TTCT**GCAG**AGTCTTCTCCGGACAGTCAT 3' |
| T08-34 KSKRSRR Reverse-4 | 5' **CGGG**AGTCTTCTCCGGACAGTCAT 3' |
| T08-34 KSKRSRR Reverse-5 | 5' G**GACG**AGTCTTCTCCGGACAGTCAT 3' |
| T08-34 KSKRSRR Reverse-6 | 5' TT**CTGA**AGTCTTCTCCGGACAGTCAT 3' |
| T08-34 KSKRSRR Reverse-7 | 5' AAC**ACTA**AGTCTTCTCCGGACAGTCAT 3' |
| T08-34 KSKRSRR Reverse-8 | 5' T**GGCA**AGTCTTCTCCGGACAGTCAT 3' |
| T08-34 KSKRSRR Reverse-9 | 5' CC**GTCT**AGTCTTCTCCGGACAGTCAT 3' |
| T08-34 KSKRSRR Reverse-10 | 5' GAT**TGTT**AGTCTTCTCCGGACAGTCAT 3' |
| T08-34 KSKRSRR Reverse-11 | 5' AGAG**TCTC**AGTCTTCTCCGGACAGTCAT 3' |
| T08-34 KSKRSRR Reverse-12 | 5' **TTTA**AGTCTTCTCCGGACAGTCAT 3' |
| T08-34 KSKRSRR Reverse-13 | 5' C**CTAC**AGTCTTCTCCGGACAGTCAT 3' |
| T08-34 KSKRSRR Reverse-14 | 5' AC**CGTA**AGTCTTCTCCGGACAGTCAT 3' |
| T08-34 KSKRSRR Reverse-15 | 5' T**ATGC**AGTCTTCTCCGGACAGTCAT 3' |
| T08-34 KSKRSRR Reverse-16 | 5' GC**TTAG**AGTCTTCTCCGGACAGTCAT 3' |
| T08-34 KSKRSRR Reverse-17 | 5' CGC**AAAC**AGTCTTCTCCGGACAGTCAT 3' |
| ^a^ The sample-specific DNA barcodes are indicated in bold. | |
