# Supplementary material for: An Experimental and Computational Evolution-Based Method to Study a Mode of Co-evolution of Overlapping Open Reading Frames in the AAV2 Viral Genome
Source: PLoS One. 2013 Jun 24;8(6):e66211. doi: 10.1371/journal.pone.0066211 (PMC3691236; doi:10.1371/journal.pone.0066211)
Supplement: Table S6 — Means and standard deviations of MW, IP and GRAVY scores of the viable VP and AAP heptapeptides. (DOCX) [file pone.0066211.s010.docx]

|  | MW | IP | GRAVY |
| --- | --- | --- | --- |
| VP (143) | 795.47 + 55.14^a^ | 5.65 + 0.93 | 1.27 + 1.05 |
| AAP (487) | 801.21 + 91.88 | 11.94 + 0.83 | -1.98 + 0.71 |

**Table S6. Means and standard deviations of MW, IP and GRAVY scores of viable VP and AAP heptapeptides.**

^a^Values represent mean + SD.

Abbreviations: MW, molecular weight; IP, isoelectric point; GRAVY, grand average of hydropathicity.
