# Supplementary material for: An Experimental and Computational Evolution-Based Method to Study a Mode of Co-evolution of Overlapping Open Reading Frames in the AAV2 Viral Genome
Source: PLoS One. 2013 Jun 24;8(6):e66211. doi: 10.1371/journal.pone.0066211 (PMC3691236; doi:10.1371/journal.pone.0066211)
Supplement: Table S7 — VP and AAP amino acid matrices used for the evolutionary algorithm. (DOCX) [file pone.0066211.s011.docx]

|  | Objective function values | | | | | | |
| --- | --- | --- | --- | --- | --- | --- | --- |
|  |  | Amino acid position | | | | |  |
| Amino acid | P1  (Q)^a^ | P2  (V) | P3  (K) | P4  (E) | P5  (V) | P6  (T) | P7  (Q) |
| A | 0.122877123 | 0.136863137 | 0.142857143 | 0.137862138 | 0.132867133 | 0.134865135 | 0.141858142 |
| C | 0.114885115 | 0.137862138 | 0.138861139 | 0.130869131 | 0.134865135 | 0.123876124 | 0.126873127 |
| D | 0.142857143 | 0.142857143 | 0.142857143 | 0.127872128 | 0.142857143 | 0.141858142 | 0.13986014 |
| E | 0.141858142 | 0.142857143 | 0.142857143 | 0.138861139 | 0.137862138 | 0.141858142 | 0.140859141 |
| F | 0.142857143 | 0.142857143 | 0.114885115 | 0.124875125 | 0.140859141 | 0.135864136 | 0.134865135 |
| G | 0.116883117 | 0.13986014 | 0.142857143 | 0.138861139 | 0.141858142 | 0.114885115 | 0.13986014 |
| H | 0.142857143 | 0.142857143 | 0.108891109 | 0.140859141 | 0.142857143 | 0.138861139 | 0.135864136 |
| I | 0.142857143 | 0.135864136 | 0.142857143 | 0.138861139 | 0.140859141 | 0.141858142 | 0.140859141 |
| K | 0.141858142 | 0.142857143 | 0.142857143 | 0.142857143 | 0.142857143 | 0.141858142 | 0.142857143 |
| L | 0.141858142 | 0.134865135 | 0.133866134 | 0.13986014 | 0.13986014 | 0.133866134 | 0.104895105 |
| M | 0.136863137 | 0.137862138 | 0.130869131 | 0.13986014 | 0.142857143 | 0.140859141 | 0.137862138 |
| N | 0.140859141 | 0.141858142 | 0.142857143 | 0.138861139 | 0.141858142 | 0.133866134 | 0.141858142 |
| P | 0.142857143 | 0.140859141 | 0.141858142 | 0.142857143 | 0.138861139 | 0.142857143 | 0.142857143 |
| Q | 0.13986014 | 0.142857143 | 0.141858142 | 0.142857143 | 0.13986014 | 0.141858142 | 0.138861139 |
| R | 0.141858142 | 0.142857143 | 0.133866134 | 0.142857143 | 0.142857143 | 0.13986014 | 0.142857143 |
| S | 0.125874126 | 0.138861139 | 0.142857143 | 0.123876124 | 0.126873127 | 0.114885115 | 0.136863137 |
| T | 0.135864136 | 0.138861139 | 0.142857143 | 0.130869131 | 0.135864136 | 0.131868132 | 0.142857143 |
| V | 0.112887113 | 0.044955045 | 0.142857143 | 0.122877123 | 0.061938062 | 0.137862138 | 0.107892108 |
| W | 0.142857143 | 0.142857143 | 0.106893107 | 0.124875125 | 0.142857143 | 0.142857143 | 0.138861139 |
| Y | 0.142857143 | 0.142857143 | 0.133866134 | 0.142857143 | 0.142857143 | 0.137862138 | 0.134865135 |

**Table S7A. VP amino acid matrix used for the evolutionary algorithm.**

^a^The amino acids in parentheses are those found in the wild type VP protein.

**Table S7B. AAP amino acid matrix used for the evolutionary algorithm.**

|  | Objective function values | | | | | | |
| --- | --- | --- | --- | --- | --- | --- | --- |
|  |  | Amino acid position | | | | |  |
| Amino acid | P1  (K)^b^ | P2  (S) | P3  (K) | P4  (R) | P5  (S) | P6  (R) | P7  (R) |
| A | 0.119976533 | 0.130536814 | 0.131710179 | 0.127603403 | 0.135816955 | 0.132883544 | 0.126723379 |
| C | 0.141977119 | 0.142270461 | 0.140510414 | 0.141683778 | 0.141097096 | 0.142270461 | 0.140217072 |
| D | 0.142563802 | 0.142270461 | 0.142563802 | 0.142563802 | 0.142270461 | 0.142857143 | 0.142857143 |
| E | 0.139923731 | 0.141683778 | 0.141683778 | 0.140510414 | 0.140217072 | 0.141097096 | 0.142563802 |
| F | 0.142563802 | 0.142857143 | 0.142857143 | 0.142270461 | 0.142857143 | 0.142563802 | 0.142270461 |
| G | 0.107069522 | 0.093575829 | 0.104722793 | 0.112643004 | 0.117043121 | 0.107069522 | 0.085068935 |
| H | 0.141390437 | 0.142270461 | 0.140217072 | 0.141683778 | 0.141683778 | 0.141683778 | 0.141683778 |
| I | 0.142857143 | 0.142857143 | 0.142857143 | 0.142563802 | 0.142857143 | 0.142857143 | 0.142857143 |
| K | 0.142563802 | 0.140803755 | 0.140510414 | 0.139923731 | 0.139337049 | 0.140217072 | 0.142563802 |
| L | 0.140803755 | 0.141390437 | 0.139923731 | 0.140803755 | 0.13963039 | 0.13963039 | 0.141097096 |
| M | 0.142270461 | 0.141977119 | 0.141977119 | 0.141977119 | 0.142270461 | 0.141977119 | 0.142270461 |
| N | 0.142857143 | 0.142563802 | 0.142857143 | 0.142857143 | 0.142270461 | 0.141977119 | 0.142270461 |
| P | 0.124083309 | 0.126136697 | 0.133176885 | 0.125550015 | 0.133470226 | 0.136403637 | 0.12936345 |
| Q | 0.138750367 | 0.141683778 | 0.140803755 | 0.139923731 | 0.139923731 | 0.140217072 | 0.141977119 |
| R | 0.103842769 | 0.102669405 | 0.092109123 | 0.104722793 | 0.075682018 | 0.083895571 | 0.113816368 |
| S | 0.140510414 | 0.139043708 | 0.136403637 | 0.133763567 | 0.135523614 | 0.138163684 | 0.13699032 |
| T | 0.13963039 | 0.139043708 | 0.139923731 | 0.136403637 | 0.140510414 | 0.138457026 | 0.138163684 |
| V | 0.137577002 | 0.137283661 | 0.136403637 | 0.134936932 | 0.13963039 | 0.138163684 | 0.137870343 |
| W | 0.140217072 | 0.140803755 | 0.140803755 | 0.13963039 | 0.139337049 | 0.13963039 | 0.141977119 |
| Y | 0.142857143 | 0.142563802 | 0.142270461 | 0.142270461 | 0.142857143 | 0.142270461 | 0.141683778 |

^b^The amino acids in parentheses are those found in the wild type AAP protein.
