# Supplementary material for: An Experimental and Computational Evolution-Based Method to Study a Mode of Co-evolution of Overlapping Open Reading Frames in the AAV2 Viral Genome
Source: PLoS One. 2013 Jun 24;8(6):e66211. doi: 10.1371/journal.pone.0066211 (PMC3691236; doi:10.1371/journal.pone.0066211)
Supplement: Table S8 — Cut-off values for sub-penalty functions. (DOCX) [file pone.0066211.s012.docx]

|  | Cut off values^a^ | |
| --- | --- | --- |
| Sub-penalty functions | Lower | Upper |
| *vp_mw_pen(x)* | 675.8 | 995.0 |
| *vp_ip_pen(x)* | 3.75 | 10.01 |
| *vp_gravy_pen(x)* | -1.857 | 3.314 |
| *aap_mw_pen(x)* | 516.5 | 1068.3 |
| *aap_ip_pen(x)* | 5.47 | 12.70 |
| *aap_gravy_pen(x)* | -3.943 | 0.943 |
| *vp12_mw_pen(x)* | 162.16 | 263.33 |
| *vp23_mw_pen(x)* | 214.28 | 317.4 |
| *vp12_gravy_pen(x)* | -1.35 | 4.35 |
| *vp23_gravy_pen(x)* | -2.4 | 4 |
| *vp34_ip_pen(x)* | 5.36 | 7.25 |

**Table S8. Cut-off values for sub-penalty functions.**

^a^If a value in a category is either lower than its lower cut-off value or higher than its upper cut-of value, "1" is assigned to the sub-penalty function in the category. Otherwise, "0" is assigned to the sub-penalty function.
