# Supplementary material for: An Experimental and Computational Evolution-Based Method to Study a Mode of Co-evolution of Overlapping Open Reading Frames in the AAV2 Viral Genome
Source: PLoS One. 2013 Jun 24;8(6):e66211. doi: 10.1371/journal.pone.0066211 (PMC3691236; doi:10.1371/journal.pone.0066211)
Supplement: Text S1 — Supporting Materials and Methods. (DOCX) [file pone.0066211.s013.docx]

**Text S1 Supporting Materials and Methods**

**Plasmid Construction**

pCMV-FLAG-cmAAP is a plasmid expressing a codon-modified version of AAV2 AAP. pCMV-FLAG-cmAAP was constructed as follows. We first created a mammalian expression plasmid vector, p4.3c, that carries the human cytomegalovirus (CMV) immediately early gene enhancer/promoter, encephalomyocarditis virus-derived internal ribosome entry site (IRES), and simian virus 40 (SV40)-derived polyadenylation signal (polyA). To construct p4.3c, pIRES was purchased from Clontech (Mountain View, CA), cut with Cla I and Nae I, and self-ligated. This procedure removed the SV40 promoter-driven neomycin resistance gene expression cassette from pIRES. Then we de novo synthesized a FLAG-tagged codon-modified AAV2 AAP ORF by PCR using the following oligonucleotides and Platinum Pfx DNA polymerase (Life Technologies, Grand Island, NY). In the following primer sequences, the Eco RI and Xba I sites used for cloning are in bold and underlined, respectively.

AAP-1 Forward:

5' ATCT**GAATTC**ACCATGGACTACAAGGACGACGATGACAAAGAAACCCAAACCCAATATCTCACACCTTCCC 3'

AAP-2 Reverse:

5' TCGGTGGGTGCTCTGGTGATGGTTTGCCACTGATGGGCGACGGCTTGCAGCCATCTGATCAGCTCCCACACGAGGGGAGGTTGTTGATGGGAATCGCTCAGGGAAGGTGTGAGATATTGGGTTTGGGTTT 3'

AAP-3 Forward:

5' GTGGCAAACCATCACCAGAGCACCCACCGAATGGGTGATCCCTAGGGAGATCGGAATCGCCATCCCTCACGGCTGGGCCACCGAAAGCAGCCCTCCTGCCCCTGAGCCCGGCCCTTGTCCTCCAACCACC 3'

AAP-4 Reverse:

5' TGTCGGTGCTGGTCAGGATGCCGCCCAGAGGGGCGGTGGCCAGGGTGGTAATGGTGGTTCTGGGTTCTTGGTTAGCGGGGAACTTATTGGTGCTTGTTGTGGTGGTTGGAGGACAAGGGCCGGGCTCAGG 3'

AAP-5 Forward:

5' CTCTGGGCGGCATCCTGACCAGCACCGACAGCACCGCTACCTTCCATCATGTCACCGGAAAGGATAGCAGCACCACCACCGGCGACAGCGATCCCAGGGATAGCACCAGCTCCAGCCTGACCTTTAAAAG 3'

AAP-6 Reverse:

5' GCTGGTTCTGCTGGATGTGCTTCTGGTGAGCAGGCATCTAAATCTGGCGGGCAGTGTGATGGGCAGTCTTCTCCGGACGGTCATCCTTCTGCTTCTTTTGCTTTTAAAGGTCAGGCTGGAGCTGGTGCTA 3'

AAP-7 Forward:

5' CTCACCAGAAGCACATCCAGCAGAACCAGCAGCGCCAGAAGAATTAAAGACGCTAGCAGAAGAAGCCAACAAACCAGCAGCTGGTGTCATAGCATGGACACAAGCCCTTAACTCTAGAGGATA 3'

AAP-8 Reverse:

5' ATCCTCTAGAGTTAAGGGCTTGTGTCCATG 3'

The resulting PCR product was inserted between Eco RI and Xba I sites of p4.3c. This resulted in construction of pCMV-FLAG-cmAAP. We designed the expression cassette so that the FLAG-tagged AAP translation was initiated by the ATG starter codon preceded by the Kozak consensus sequence, CACC. The AAP codon modification was made for optimal expression in mammalian cells and preventing recombination between the AAP ORF DNA supplied in trans and AAV viral genomes carrying the unmodified version of the AAP ORF DNA.

pAAV2R585E-RepVP3, which expresses all the Rep proteins, VP3 protein carrying R585E mutation, but does not express VP1, VP2 or AAP, was constructed in the following manner. A pUC118-based plasmid carrying the entire AAV2 genome with R585E mutation, pUC118-AAV2R585E-SBBXEB-PBS, was de novo synthesized by Takara Bio Inc. (Otsu, Shiga, Japan). This plasmid has multiple silent mutations in the VP1 ORF to make new restriction enzyme recognition sites. Such silent mutations include C3213T and C3216T in the AAV2 genome (NC001041) that makes a new Spe I site. Using PCR-mediated site-directed mutagenesis techniques, we introduced following mutations: (1) We destroyed the VP1 starter codon ATG by changing it to ACT to prevent VP1 translation; (2) an AAV2 genome segment spanning from nucleotide positions 2254 to 2808, which corresponds to the region between the Rep 40/68 stop codon and the VP3 starter codon, was removed; (3) An additional TAA stop codon that terminates translation from the VP1 starter codon was inserted just downstream of the Rep 40/68 stop codon; and (4) Kozak consensus sequence was introduced for the VP3 starter codon. The third and fourth modifications were attained by inserting the following nucleotides, ATAATCACC, between the TAA stop in Rep 40/68 ORF and the ATG starter in VP1 ORF. We confirmed that the above modifications preserve VP3 expression but abolish the AAP functions. This was evidenced by the observation that pAAV2R585E-RepVP3 could and could not make VP3 only particles in the presence and absence of AAP, respectively. To make pAAV2-RepVP3, the R585E mutation was changed to the wild type by PCR-mediated site-directed mutagenesis.

pAAV2-RepVP3-Lib-BB is the backbone plasmid into which we inserted a library of 21-base pair double-stranded oligonucleotides translated into random heptapeptides in place of QVKEVTQ. We first constructed pAAV2R585E-RepVP3-Lib-BB in the following manner. We introduced one silent mutation T1886C in the Rep ORF and another silent mutation C3147T in the VP frame to destroy and create Hind III sites, respectively. In addition, there were two silent mutations, C3213T and C3216T, which create a new Spe I site as described earlier. Then a double-stranded oligonucleotide, (Hind III)AAGCTTAGTTTAAACTGACTAGT(Spe I), was inserted between the Hind III and Spe I sites in place of the native sequence. This replacement resulted in the introduction of three stop codons, TAG, TAA and TGA, in all the three frames to prevent expression of viable VP3 protein. Finally, the R585E mutation was converted to its wild type to create pAAV2-RepVP3-Lib-BB.

pAAV2-RepVP3-Lib-0 is the plasmid library with which we produced AAV2-RepVP3-Lib-1 virus library carrying random heptapeptides in place of QVKEVTQ in the VP3 protein. Construction of the pAAV2-RepVP3-Lib-0 plasmid library was done with the following oligonucleotides purchased from Sigma-Aldrich (St. Louis, MO). The Hind III and Spe I sites used for cloning are in bold and underlined, respectively.

70-98 VP 7-mer Forward:

5' CTAA**AAGCTT**TTCAACATC(NNK)_7_AATGACGGTACGACGACGATTGCCAATAACCTTACTAGT 3' (K=G or T)

70-98 VP 7-mer Reverse:

5' TCTTACTAGTAAGGTTATTGGCAA 3'

Oligonucleotide annealing, double-stranded oligonucleotide synthesis and plasmid library construction were performed as previously described [[22](#_ENREF_21)]. The Hind III-Spe I fragment of the double-stranded oligonucleotide was inserted between the corresponding sites in pAAV2-RepVP3-Lib-BB to create the pAAV2-RepVP3-Lib-0 plasmid library. The diversity of this library estimated by a colony counting method was 2x10^6^.

pAAV2-CMV-cmAAP is a plasmid with which we produced an AAV2 vector expressing cmAAP. We PCR-amplified the cmAAP ORF with Kozak consensus sequence but without the FLAG sequence using pCMV-FLAG-cmAAP as a template. This procedure also introduced Eco RI and Hind III sites for cloning. Next, the Eco RI-Hind III PCR fragment was inserted between the corresponding restriction enzyme recognition sites in pAAV-MCS (Agilent, Santa Clara, CA), which is a single-stranded AAV vector plasmid designed for CMV promoter-driven transgene expression. The resulting construct was pAAV2-CMV-cmAAP. In this plasmid, the cmAAP ORF is placed between the CMV enhancer-promoter/human β-globin intron and the human growth hormone polyadenylation signal.

pAAV2-CMV-cmAAP-Lib-0 is the plasmid library with which we produced AAV2-CMV-cmAAP-Lib-1 virus library carrying random heptapeptides in place of KSKRSRR in the AAP protein. Construction of pAAV2-CMV-cmAAP-Lib-0 is as follows. We first created pAAV2-CMV-cmAAP-Lib-BB, which served as a backbone plasmid for random oligonucleotide insertion, by replacing AAV2 nucleotide sequence from 3137 to 3191 with ACTAGTAGTTTAAACTGA**TCCGGA**, in which Spe I and Bsp EI sites are underlined and in bold, respectively. This manipulation removed the sequence coding KSKRSRR, introduced silent mutations to create Spe I and Bsp EI sites, and introduced three stop codons; TAG, TAA and TGA, in all three frames to prevent expression of viable AAP protein. Then, double-stranded oligonucleotides that code random heptapeptides in place of KSKRSRR and their adjacent amino acids, were inserted in-frame between Spe I and Bsp EI sites in the same manner as described above. This resulted in the creation of pAAV2-CMV-cmAAP-Lib-0. The following oligonucleotides (Sigma-Aldrich) were used to make the plasmid library. The diversity of this library estimated by a colony counting method was 2x10^6^.

T07-108 AAP 7-mer Forward:

5' AGGGATAGCACTAGTTCAAGCCTGACCTTC(NNK)_7_ATGACTGTCCGGAGAAGACTG 3' (K=G or T)

T07-108 AAP 7-mer Reverse:

5' CAGTCTTCTCCGGACAGTCAT 3'

The production of AAV2-CMV-cmAAP-Lib viral particles in the study required a non-standard AAV helper plasmid, pHLP-RepVP3, which provides Rep and VP3 proteins but is devoid of AAP expression. This plasmid was constructed by transferring the AAV2-RepVP3 viral genome sequence except for the two AAV inverted terminal repeats (ITR) from pAAV2-RepVP3 to pBluescript II KS(-) (Stratagene, La Jolla, CA). pHLP22-R585E is a derivative of pAAV-RC (Cell Biolabs, Inc. San Diego, CA), and has the de novo synthesized AAV2R585E genome sequence with multiple silent mutations in place of the original AAV2 genome sequence contained in pAAV-RC. pHLP22 is the pHLP22-R585E-derived plasmid in which the R585E mutation has reverted to the wild type. All the AAV helper plasmids carrying a VP mutation were constructed using pHLP22 as a platform. An adenovirus helper plasmid, pHelper, was purchased from Agilent. Bacterial transformation with the plasmids used in this study was done using *Escherichia coli* ElecrtoMax DH10B (Life Technologies) except for pAAV2-CMV-cmAAP-Lib-BB, for which we used C2925 (New England BioLabs (NEB), Ipswich, MA) due to dam methylation of the Bsp EI site we used for cloning.

The 21-nucleotide-long random sequence regions in the plasmid libraries were extensively characterized by Illumina sequencing. The method for Illumina sequencing is described below. The sequencing analysis of pAAV2-RepVP3-Lib-0 and pAAV2-CMV-cmAAP-Lib-0 identified 1,106,807 and 662,265 different in-frame peptide insertions, among which 935,590 and 559,524 had no stop codons in the VP3 and AAP libraries, respectively. Therefore, the frequencies of peptide insertions with one or more stop codons were 15.4% and 7.1% for the VP3 and AAP libraries, respectively. Of the peptide insertions with no stop codons, 930,372 (99.4%) and 554,743 (99.1%) in the VP3 and AAP libraries, respectively, were heptapeptides, and others were either longer or shorter than heptapeptides.

**Experimental directed evolution.**

AAV2-RepVP3-Lib and AAV2-CMV-cmAAP-Lib virus libraries were produced by a 1-step method to minimize the phenotype-genotype dissociation problem in the libraries [22]. Briefly, we produced the AAV viruses by transfecting HEK293 cells with 15 ng of the AAV library plasmid and 15 μg each of helper plasmids per 15 cm dish (please refer to **Table S4**). In this method, the quantity of AAV library plasmid DNA to be transfected was substantially reduced compared to the quantity of helper plasmids. In our preliminary experiment, we found that a 1000-fold reduction of the quantity of AAV library plasmid could still produce a sufficient amount of viral particles usable in the directed evolution experiment. Forty-eight hours post-transfection of plasmid DNA, cells were harvested and the AAV2-RepVP3-Lib-1 and AAV2-CMV-cmAAP-Lib-1 viral particles were collected from the cells by three cycles of freezing and thawing. The crude lysates were treated with Benzonase (MERCK KGaA, Darmstadt, Germany) and purified by CaCl_2_ precipitation followed by polyethylene glycol (PEG) 8000 precipitation as described elsewhere [[36](#_ENREF_36)]. Subsequently, the viral preparation was treated with DNase I (Roche, F. Hoffmann-La Roche, Ltd., Basel, Switzerland) followed by Proteinase K (Life Technologies).

The viral genome DNA was purified by phenol / chloroform extraction and recovered by ethanol precipitation and used as a template for PCR with Platinum Pfx DNA polymerase to amplify the random 21 nucleotide-long region. The PCR-amplified fragments were cloned into either pAAV2-RepVP3-Lib-BB or pAAV2-CMV-cmAAP-Lib-BB as described above and the second round plasmid libraries, pAAV2-RepVP3-Lib-1 and pAAV2-CMV-cmAAP-Lib-1, were created. This cycle was repeated three times until we obtained AAV2-RepVP3-Lib-3 and AAV2-CMV-cmAAP-Lib-3 viral particles. The primers used for the PCR amplification were as follows.

70-98 VP Forward:

5’ TCAACTTCAAGCTTTTCAACATC 3’

70-98 VP Reverse:

5’ CGAGTCAGTAAACACCTGAACCGT 3’

T07-108 AAP Forward:

5’ CAGGGATAGCACTAGTTCAAGC 3’

T07-108 AAP Reverse:

5’ AGTCTTCTCCGGACAGTCAT 3’

**Illumina sequencing**

At each round of selection, the random 21 nucleotide-long region of the AAV genome corresponding to the QVKEVTQ/KSKRSRR was PCR-amplified using the primers described in **Table S5**. Each primer contains a sample-specific 3 or 4 nucleotide-long DNA barcode and frame-shifting 1 to 5 nucleotides at the 5' end in addition to the 20 nucleotide-long AAV genome-specific sequence. The DNA barcodes were incorporated for multiplexed sequencing [[37](#_ENREF_37)] and a frame-shifting technique was used to overcome the issue of low sequence diversity of PCR products in reference image construction [[23](#_ENREF_22)]. We amplified the random 21 nucleotide-long region in the original plasmid libraries and the AAV viral genomes recovered from each directed evolution cycle. For plasmid library amplification, we used 10 ng of plasmid DNA as a template, while for the viral genome amplification, we used one-twenty fifth of the AAV viral genome DNA recovered from a 15 cm dish. PCR cycles were the following: 2 min at 95°C, 35 cycles of 15 s at 95°C and 30 s at 68°C, and subsequently 5 min at 68°C. The PCR products are in a range of 69 to 75 bp. Up to 17 PCR products carrying different sample-specific DNA barcodes were mixed together and subjected to 100-cycle single-end Illumina sequencing using an Illumina HiSeq 2000 according to the manufacturer's recommendation. An algorithm for binning sequence reads by sample-specific DNA barcodes, extraction of the sequences at the random 21 nucleotide-long region, and their translation into amino acids, was developed and implemented in Perl at the Pittsburgh Supercomputing Center.

**Cell culture experiments**

The VP3 only viral capsid formation analysis was done in the following manner. HEK293 cells were seeded in 6 cm dishes at a density of 1x10^6^ cells. Twenty-four hours later, the cells were transfected with 4 μg each of the plasmids required for AAV2-RepVP3 or AAV2-CMV-cmAAP virus production (**Table S4**) in the same way as that for the standard AAV vector production [[36](#_ENREF_36)]. Transfected cells were harvested 48 hours post-transfection, and the viral particles were collected and purified by CaCl_2_ precipitation followed by PEG 8000 precipitation as described above. After DNase I and Proteinase K treatment, the viral genome DNA was purified by phenol / chloroform extraction and ethanol precipitation, and quantified by a quantitative dot blot assay using a ^32^P-labeled *rep* gene or CMV promoter DNA probe. The experiments were done in triplicate and the results were expressed as percentage of the virus production yield relative to that of the wild type controls.

For the virus infectivity assay, we produced dsAAV2-CMV-GFP virions containing all VP1, VP2 and VP3 proteins as described above. HEK293 cells seeded in 24-well plates were infected with wild type dsAAV2-CMV-GFP or its capsid mutants at a multiplicity of infection (MOI) of 20,000 for 1 hour. Forty-eight hours post-infection, cells were observed under an EVOS inverted fluorescence microscope and underwent flow cytometric analysis using a FACSCalibur (Becton Dickinson, Franklin Lakes, NJ). At least 5000 cells were counted in the flow cytometry. For the experiment investigating infectivity of the AAV2 K321A mutant, recombinant AAV crude lysates were used to infect HEK293 cells at 32, 37 or 39.5 °C as Wu et al. did so in their study investigating the heat-sensitive AAV2 K321A/E322A mutant [[28](#_ENREF_27)]. The virus infection experiments were done in triplicate or quadruplicate.

**Computational directed evolution**

In the evolutionary algorithm for directed evolution of heptapeptides in silico, we defined a fitness function as the sum of an objective function term and a penalty function term, and applied a linear ranking selection scheme [[38](#_ENREF_38)]. The objective function is the sum of the following 8 sub-objective functions; *vp_mw_obj(x), vp_ip_obj(x), vp_gravy_obj(x), vp_aa_obj(x), aap_mw_obj(x), aap_ip_obj(x), aap_gravy_obj(x),* and *aap_aa_obj(x)*  ( *x* ∈ *{x_1_*, *x_2_*, ... *x_N_}*, where *x_i_ is* a 22 nucleotide-long DNA sequence and *N* is the number of 22 nucleotide-long DNA sequences in a population). In *x_i_* , the first 21 nucleotides code VP ORF, which is translated into a VP heptapeptide (*vp_7mer(x_i_)* ), and the +1 frame-shifted 21 nucleotides code AAP ORF, which is translated into an AAP heptapeptide ( *aap_7mer(x_i_)* ). Each sub-objective function is continuous and takes values from "0" up to "1", where "0" and "1" represent the best and worst fits, respectively. As for the penalty term, we defined the penalty function as the sum of a total of 11 sub-penalty functions; *vp_mw_pen(x), vp_ip_pen(x), vp_gravy_pen(x), aap_mw_pen(x), aap_ip_pen(x), aap_gravy_pen(x),* *vp12_mw_pen(x), vp23_mw_pen(x), vp12_gravy_pen(x), vp23_gravy_pen(x), and vp34_ip_pen(x)* ( *x* ∈ *{x_1_*, *x_2_*, ... *x_N_}* ). The penalty function was added to the objective function term so that constraint violation imposes a high cost on the fitness function. Each sub-penalty function can have only one of two values "0" and "1" for *x_i_* ( *_i_* ∈ *{1, 2, ... N}* ) with no violation and with violation, respectively.

The definition of sub-objective functions in the evolutionary algorithm used in the study is as follows. *vp_mw_obj(x)* and *aap_mw_obj(x)* provide values indicating how well the VP and AAP heptapeptides translated from a given 22 nucleotide-long DNA sequence "*x_i_*" (*i.e.*, *vp_7mer(x_i_)* and *aap_7mer(x_i_)*, respectively) fit to the distribution of the molecular weights of the viable VP and AAP heptapeptides identified in the experimental evolution experiment. We assumed that if *vp_mw(x_i_)*, which is the molecular weight of *vp_7mer(x_i_)*, is the same as the mean of the molecular weights of the experimentally identified capsid-forming 143 VP heptapeptides (*i.e.*, *vp143_mw_mean*), *vp_7mer(x_i_)* fits most comfortably to the biochemical property in the aspect of molecular weight; therefore we assign a "0" value to *vp_mw_obj(x_i_)*. For other *vp_mw(x_i_)* values, we applied z-normalization using *vp143_mw_mean* value and the standard deviation of the molecular weights of the 143 VP heptapeptides (*i.e.*, *vp143_mw_sd*), where the *z_score_vp_mw(x_i_),* which is a modified z-score of *vp_mw(x_i_)*, can be calculated in the following equation: *z_score_vp_mw(x_i_)* = |*vp_mw(x_i_)* - *vp143_mw_mean|* / *vp143_mw_sd*. Although *z_score_vp_mw(x_i_)* is not the true z-score and takes only values between 0 and 1, it provides a value indicating how much a given value is deviated from the mean. Then a probability given by each *z_score_vp_mw(x_i_)* is used to calculate each sub-objective function. In the study, we define *vp_mw_obj(x_i_)* as the function formulated as *vp_mw_obj(x_i_)* = 1- 2 x *uprob* ( *z_score_vp_mw(x_i_)* ), where uprob ( *z_score_vp_mw(x_i_)* ) provides upper probability when z-score takes a value of *z_score_vp_mw(x_i_)*. For example, when *vp_mw(x_i_)* equals to the mean, *z_score_vp_mw(x_i_)* is "0"; therefore, *vp_mw_obj(x_i_)* is "0". When *vp_mw(x_i_)* is deviated from the mean by 1.96 standard deviations, *z_score_vp_mw(x_i_)* is "1.96"; therefore, *vp_mw_obj(x_i_)* is "0.95". We defined the other 5 objective functions, *vp_ip_obj(x), vp_gravy_obj(x), aap_mw_obj(x), aap_ip_obj(x),* and *aap_gravy_obj(x)*, in the same manner. The mean values and standard deviations of molecular weights, isoelectric points and GRAVY scores of the 143 VP and 487 AAP heptapeptides are summarized in **Table S6**. Five of the experimentally identified functionally competent 492 AAP heptapeptides were excluded from the data transformation procedure because they were outliers. As for the sub-objective functions for the amino acid compositions in the VP and AAP heptapeptides (*i.e.*, *vp_aa_obj(x)* and *aap_aa_obj(x)* ), we created amino acid composition matrices based on the actual data obtained by the experimental evolution experiments (**Table S7**). We assigned a value of "1/7" to each of the 7 amino acid positions in the VP and AAP heptapeptides. At each amino acid position, we assigned "0" and "1/7" values to the amino acid residues that are found most frequently and those that are not found, respectively. As for the other amino acid residues at each position, a value was assigned to each amino acid residue in such a way that the value is linearly proportional to the probability of the absence of the amino acid residue at the position.

In each sub-penalty function, "1" was given to outliers. The outliers were those that showed values that were found outside the range between the smallest and largest values observed in the experimental evolution experiment. In each penalty function, we defined cut-off values as summarized in **Table S8**. *vp_mw_pen(x), vp_ip_pen(x), vp_gravy_pen(x), aap_mw_pen(x), aap_ip_pen(x)* and *aap_gravy_pen(x)* are the sub-penalty functions for molecular weights, isoelectric points and GRAVY scores of the VP and AAP heptapeptides. *vp12_mw_pen(x), vp23_mw_pen(x), vp12_gravy_pen(x), vp23_gravy_pen(x), and vp34_ip_pen(x)* are the additional 5 sub-penalty functions that concern the biochemical properties of P1and P2 (*vp12*), P2 and P3 (*vp23*) and P3 and P4 (*vp34*) amino acids in the VP heptapeptide.

In the co-evolutionary algorithm, the objective function was weighted evenly between VP and AAP sub-objective functions because neither VP nor AAP is dispensable for capsid formation. However, the constraints of the amino acid compositions in the VP heptapeptide were found stronger than those in the AAP heptapeptide. Therefore, we performed an experiment to determine *p_vp_aa,* which is a parameter that determines the weight of *vp_aa_obj(x).* Using a data set of 930372 different VP heptapeptides found in the initial pAAV2-RepVP3-Lib plasmid library (pAAV2-RepVP3-Lib-0) and the viable 143 VP heptapeptides, we calculated the VP fitness function ( *f_vp(x)* ) values of each heptapeptide using the following formula,

*f_vp(x)=vp_mw_obj(x)+vp_ip_obj(x)+vp_gravy_obj(x)+p_vp_aa* x *vp_aa_obj(x)*

*+vp_mw_pen(x)+vp_ip_pen(x)+vp_gravy_pen(x)+vp12_mw_pen(x)+vp23_mw_pen(x)+vp12_gravy_pen(x)+vp23_gravy_pen(x)+vp34_ip_pen(x)* (*p_vp_aa ∈ {0, 1, 2, 3}*).

Then we analyzed the amino acid compositions of the VP heptapeptides in the library that showed *f_vp(x)* values within the range of the *f_vp(x)* values of the top 50 ranked viable VP heptapeptides. The characteristic pattern of the amino acid compositions found in the viable VP heptapeptides became clear when the *p_vp_aa* was 2 or 3, but was not clear when the *p_vp_aa* was 0 or 1. The same experiment was done for *p_aap_aa,* which is a parameter that determines the weight of *aap_aa_obj(x)*, by calculating the AAP fitness function ( *f_aap(x)* ) values of 554743 different AAP heptapeptides found in the initial pAAV2-CMV-cmAAP-Lib plasmid library and the viable 487 AAP heptapeptides. *f_aap(x)* follows the following formula,

*f_aap(x)=aap_mw_obj(x)+aap_ip_obj(x)+aap_gravy_obj(x)+p_aap_aa* x *aap_aa_obj(x)+aap_mw_pen(x)+aap_ip_pen(x)+aap_gravy_pen(x).*

The characteristic pattern of the amino acid compositions found in the viable AAP heptapeptides was not clear at *p_aap_aa =*0, but became appreciable when the *p_aap_aa* was 1, 2 or 3. Based on these observations, we used 2 and 1 for *p_vp_aa* and *p_aap_aa*, respectively. Thus, the fitness functions we used for single VP and AAP evolutions ( *f_vp(x)* and *f_aap (x)* ) and co-evolution ( *f_vpaap(x)* ) are formulated in the following forms where *p_vp_aa* = *2*  and  *p_aap_*aa = 1.

*f_vp(x)=( vp_mw_obj(x)+vp_ip_obj(x)+vp_gravy_obj(x)+p_vp_aa* x *vp_aa_obj(x) )* x (*p_aap_aa+3)/4*

*+(vp_mw_pen(x)+vp_ip_pen(x)+vp_gravy_pen(x)+vp12_mw_pen(x)+vp23_mw_pen(x)+vp12_gravy_pen(x)+vp23_gravy_pen(x)+vp34_ip_pen(x))*

*f_aap(x)=( aap_mw_obj(x)+aap_ip_obj(x)+aap_gravy_obj(x)+p_aap_aa* x *aap_aa_obj(x) )* x (*p_vp_aa+3)/4*

*+(aap_mw_pen(x)+aap_ip_pen(x)+aap_gravy_pen(x)).*

*f_vpaap(x)= f_vp(x) + f_aap(x)*

In the computational evolution procedure, the size of a population was fixed to 200 individuals. This population size was empirically determined and provides a sufficient search space explorable in a reasonable runtime. The initial population was created from a single ancestral DNA by introducing one random nucleotide substitution at random positions that did not cause a nonsense mutation, which is referred to as a non-nonsense mutation in this paper. The evolution followed a linear ranking selection scheme [[38](#_ENREF_38)] with a non-nonsense mutation rate of 0.5 and without a crossover. In this scheme, the 200 individuals in a population were ranked based on their fitness function and selected for 200 reproduction events at a probability linearly proportional to the fitness rank of each individual. Then each selected individual reproduced one progeny to which one non-nonsense mutation was introduced randomly at a probability of 0.5. In this selection procedure, all the individuals had a chance to reproduce a progeny to be included in the next generation in such a way that the most and least fit individual had the most and least chance to reproduce. We repeated this procedure until the 200th generation was reached in 200 different populations. All the 40,000 individuals in the 200th generation were subjected to the amino acid composition and biochemical analyses. For the co-evolution analysis of xVKDVxx and xVKEVxx motifs, 200 ancestral sequences coding xVKDVxx or xVKEVxx were generated, and subjected to the same computational co-evolution procedure except that the non-nonsense mutations to be introduced in progeny were restricted to only those that code xVK(D/E)Vxx VP sequences. Evolutionary algorithms and characterization of biochemical properties of heptapeptides were implemented in Perl with BioPerl and CPAN modules.

**Bioinformatics**

GenBank accession numbers for the 128 AAV species used in the study are AF028704, AF028705, AY186198, AY242997 to AY243023, AY349010, AY382884, AY382890, AY382891, AY530553 to AY530629, AY631965, AY631966, DQ335246, DQ813647, EU088101, EU088102, EU285562, JF926696, NC_001041, NC_001701, NC_001729, NC_001829.1, NC_002077, NC_005889, NC_006152, NC_006260 and NC_006261. The nucleotide sequences were translated using the VP ORF and AAP ORF, and the VP and AAP amino acid sequences were aligned with ClustalW [[41](#_ENREF_41)]. In the secondary structure prediction procedure using the Discrimination of Secondary Structure Class (DSC) algorithm [[27](#_ENREF_26)], the regions of 151 amino acids in length between W247 and Y397 in the VP mutants and between I54 and P204 in the AAP mutants were used to predict the secondary structure of each residue in the VP and AAP heptapeptide motifs. For simulation studies, we computationally generated 10000 random heptapeptides.

To search heptapeptide regions that can accommodate the AAP KSKRSRR function at a high probability throughout the +1 frame-shifted VP1 ORF, we unambiguously back-translated the entire 2.2-kb VP1 ORF using an algorithm implemented in Python. To this end, we back-translated the first octapeptide of the VP1 protein (*i.e.*, VP1 amino acid positions from 1 to 8) into all possible 24 nucleotide-long DNA sequences encoding the octapeptide. Then we translated all the DNA sequences from the positions 2 to 22 into heptapeptide in the AAP frame, determined their molecular weights, isoelectric points, GRAVY scores, and the amino acid compositions, and calculated their fitness function ( *f_aap(x)* ) scores. We moved this 8-amino acid sliding window by one amino acid from the N-terminus to C-terminus to scan the entire VP1 ORF and obtain information about the biochemical properties of overlapping AAP heptapeptides and their *f_aap(x)* scores.

**References**

22. Adachi K, Nakai H (2010) A New Recombinant Adeno-Associated Virus (AAV)-Based Random Peptide Display Library System: Infection-Defective AAV1.9-3 as a Novel Detargeted Platform for Vector Evolution. Gene Ther Regul 5: 31-55.

23. Bentley DR, Balasubramanian S, Swerdlow HP, Smith GP, Milton J, et al. (2008) Accurate whole human genome sequencing using reversible terminator chemistry. Nature 456: 53-59.

27. King RD, Sternberg MJ (1996) Identification and application of the concepts important for accurate and reliable protein secondary structure prediction. Protein Sci 5: 2298-2310.

28. Wu P, Xiao W, Conlon T, Hughes J, Agbandje-McKenna M, et al. (2000) Mutational analysis of the adeno-associated virus type 2 (AAV2) capsid gene and construction of AAV2 vectors with altered tropism. J Virol 74: 8635-8647.

36. Grimm D, Zhou S, Nakai H, Thomas CE, Storm TA, et al. (2003) Preclinical in vivo evaluation of pseudotyped adeno-associated virus vectors for liver gene therapy. Blood 102: 2412-2419.

37. Craig DW, Pearson JV, Szelinger S, Sekar A, Redman M, et al. (2008) Identification of genetic variants using bar-coded multiplexed sequencing. Nat Methods 5: 887-893.

38. Baker JE (1985) Adaptive selection methods for genetic algorithms. In: J. J. Grefen-stette editors. Proceedings of the First International Conference on Genetic Algorithms, Lawrence Erlbaum Associates. pp. 101-111.

41. Larkin MA, Blackshields G, Brown NP, Chenna R, McGettigan PA, et al. (2007) Clustal W and Clustal X version 2.0. Bioinformatics 23: 2947-2948.
